# New species and host records of New World, mostly Neotropical, opiine Braconidae (Hymenoptera) reared from flower-infesting, stem-galling, and stem-mining Tephritidae (Diptera)

**DOI:** 10.3897/zookeys.349.5914

**Published:** 2013-11-13

**Authors:** Robert Wharton, Allen L. Norrbom

**Affiliations:** 1 Department of Entomology, Texas A&M University, College Station, Texas 77843 USA; 2 Systematic Entomology Laboratory, USDA, ARS, c/o National Museum of Natural History, P.O. Box 37012, MRC 168, Washington, DC 20013-7012, USA

**Keywords:** Parasitoid, wasp, fly, Asteraceae, classification

## Abstract

New host records (all members of the family Tephritidae) are presented for 14 newly described species of opiine Braconidae from the neotropics and two previously described species, one from the neotropics and one from the Nearctic Region. *Doryctobracon anneae* Wharton, *Opius baderae* Wharton, *O. baeblus* Wharton, *O. cablus* Wharton, *O. dablus* Wharton, *O. danielsae* Wharton, *O. gabriellae* Wharton, *O. godfrayi* Wharton, *O. marshi* Wharton, *O. nablus* Wharton, *O. pipitae* Wharton, *O. stecki* Wharton, *O. taramegillae* Wharton, and *O. yoderi* Wharton are newly described. Hosts are newly recorded for the previously described species *Opius nympha* Fischer and *O. peleus* Fischer. A key is presented to Opiinae that have been reared from flower, stem, and leaf feeding tephritids in the New World. Host and host plant associations are discussed; a few of the tephritid host plant records are also new. *Opius cosa* (Fischer), is a **comb. n.**

## Introduction

The braconid subfamily Opiinae is comprised of koinobiont endoparasitoids of cyclorrhaphous Diptera that oviposit in the host egg or larva and emerge as adults from the host puparium. The best known and most thoroughly studied species are parasitoids of fruit-infesting Tephritidae, leaf-mining Agromyzidae, and leaf-mining Anthomyiidae (as summarized by Fischer 1971, 1972, 1977, Wharton 1997a, b, 2009, Wharton et al. 2012, Wharton and Yoder 2012, Yu et al. 2012). The species of Opiinae that attack flower-infesting and gall-making tephritids are relatively poorly known, with several published records from the Old World (reviewed by Wharton 2009), including adventive species of fruit-fly parasitoids that occasionally attack gall-formers purposefully introduced for weed biocontrol in Hawaii (Clancy 1950, Duan et al. 1997, Duan and Messing 1999). Comparable published records for the New World are lacking. The primary purpose of this work is to describe several species reared from New World stem-mining, gall-making, and flower-infesting Tephritidae. The material described below, consisting of representatives from several different species groups within *Opius* Wesmael s.l., considerably expands our understanding of the diversity of this host-parasitoid association.

Throughout much of the 1900s, most Opiinae were placed in the genus *Opius*, which eventually encompassed over 1000 species (Fischer 1971). Fischer (1972) facilitated work on the Opiinae by presenting a classification in which several distinctive genera were recognized and an extensive subgeneric classification was proposed for *Opius*. *Opius* has been subsequently reduced by removal of putatively monophyletic taxa such as *Utetes* Foerster and *Psyttalia* Walker (Wharton 1987, 1988), and by an attempt to restrict the definition of *Opius* to species with a basal lobe on the mandible (van Achterberg and Salvo 1997, Li et al. 2013). Fischer (1999), Tobias (1977, 1998), Wharton (1983, 1987, 1988, 1997a, 2006), van Achterberg (1997, 2004), Belokobylskij et al. (2003), and Wharton et al. (2012) continue to modify the classification proposed by Fischer (1972, 1977, 1987), resulting in some instability in the usage of several of the genus group names. Further delimitation of monophyletic groups is essential for progress in understanding the relationships and evolutionary biology of this large and important group of dipteran endoparasitoids. The approach taken here is that followed by Wharton (1997a, b, 2006) and Wharton et al. (2012), as modified from Fischer (1972, 1999). Thus, the species treated below are placed in *Opius* s.l. rather than *Phaedrotoma* Foerster as advocated by van Achterberg and Salvo (1997), Li et al. (2013), and to some extent Yu et al. (2012) since *Phaedrotoma* as defined by van Achterberg and Salvo (1997) is not monophyletic: it is the place-holder for species not clearly defined by derived character states. Li et al. (2013) revised the characterization of *Phaedrotoma* and also treated *Rhogadopsis* Brèthes as valid. Unfortunately, several New World taxa are intermediate between *Rhogadopsis* and *Phaedrotoma* as delineated by Li et al. (2013) for the Chinese fauna. *Phaedrotoma* is thus still problematic and shifting the bulk of the opiines of doubtful monophyly from *Opius* s.l. to *Phaedrotoma* would not serve stability. *Opius* s.s. as redefined by van Achterberg and Salvo (1997) is readily accommodated in the classification used here.

## Materials and methods

**Specimens.** Reared material of several species, including all those newly described below, was kindly sent for study to the senior author by Paul Marsh and Allen Norrbom (USDA Systematic Laboratory, Washington, D. C.), and Gary Steck (Florida Department of Agriculture and Consumer Services, Division of Plant Industry, Gainesville, Florida, FSCA). Other specimens used in this study, including type material of previously described species, were borrowed from or examined at the following institutions: American Entomological Institute, Gainesville, Florida, USA (AEIC), California Academy of Sciences, San Francisco, California, USA (CAS), Canadian National Collection, Ottawa, Ontario, Canada (CNC), Naturhistorisches Museum Wien, Vienna, Austria (NHMW), Texas A&M University Insect Collection, College Station, Texas, USA (TAMU), and National Museum of Natural History, Smithsonian Institution, Washington, D. C., USA (USNM).

In the material examined section under each species description, we record label data for the holotype exactly as they appear on the labels. We use a more standardized format for paratypes, additional specimens examined, and published data for other specimens.

**Figures.** Images were acquired digitally using either Syncroscopy’s AutoMontage® software or Helicon Focus®, in combination with a ProgRes 3008 digital camera mounted on a Leica MZ APO dissecting microscope. All images were further processed using various minor adjustment levels in Adobe Photoshop® such as image cropping and rotation, adjustment of contrast and brightness levels, color saturation, and background enhancement. Compiled images, including many not incorporated here, are available in color and high resolution at http://mx.speciesfile.org/projects/8/public/otu_group/show/386.

**Database management, digital dissemination, and ontology reference.** Illustrations and free-text diagnoses for morphospecies were assembled in mx, a web-based content management system that facilitates data management and dissemination for taxonomic and phylogenetic works (e.g., Yoder et al. 2006). The mx project is open source, with code and further documentation available at http://sourceforge.net/projects/mx-database/. Data pertinent to this work, including images, diagnoses, and descriptions, are available at http://mx.speciesfile.org/projects/8/public/otu_group/show/386.

Morphological terms used in this revision were matched to the Hymenoptera Anatomy Ontology (HAO, Yoder et al. 2010, Seltmann et al. 2012). Identifiers (URIs) in the format http://purl.obolibrary.org/obo/HAO_XXXXXXX represent anatomical concepts in HAO version http://purl.obolibrary.org/obo/hao/2011-05-18/hao.owl, as used by Wharton et al. (2012, appendix). The URIs are provided to enable readers to confirm their understanding of the anatomical structures being referenced. To find out more about a given structure, including images, references, and other metadata, use the identifier as a web-link, or use the HAO:XXXXXXX (note colon replaces underscore) as a search term at http://glossary.hymao.org. Terminology as linked through the HAO (Wharton et al. 2012, appendix) largely follows Sharkey and Wharton (1997), with a few additions from Walker and Wharton (2011) and Wharton et al. (2012). See also the useful paper on skeletal morphology of two species of Opiinae by Karlsson and Ronquist (2012) for some alternative terminology.

Quantitative data in descriptions are based on 5 individuals of each sex for the few species with longer series, and on all available material for the remaining species. Measurements largely follow Walker and Wharton (2011). Mesosomal width is the distance across the mesoscutum between the tegula; mesosomal length is the maximum distance between the dorsal curve of the anterior declivity of the mesoscutum and the ventral carina of the metapleuron. Width of the clypeus was measured at the lateral margin, which usually coincided with the middle of the anterior tentorial pit in the species of *Opius* s.l. Body length varies with state and manner of preservation, but is provided as an approximation of size along with wing and mesosomal lengths, which are better proxies for size. Ovipositor length is treated here as an important species-level character. Where more than one female was available, the ovipositor and ovipositor sheath were usually dissected for a more accurate measure of length. Otherwise, total length was estimated, and this is indicated in the descriptions by use of the word “approximately.” Quantitative data are reported to the nearest 0.05.

Plant names were checked in Tropicos (www.Tropicos.org). Host fly names were verified by the junior author.

## Results and discussion

**Biology.** Three species groups of *Opius* s.l., each with distinctive host associations, are described below: the *baderae* species group, based on *Opius baderae* Wharton, sp. n., the *godfrayi* species group, based on *Opius godfrayi* Wharton, sp. n, and the *pipitae* species group, based on *Opius pipitae* Wharton, sp. n. Within the *baderae* species group, there are two subgroups represented by different head color patterns. One group attacks tephritids in stems while the other attacks tephritids in flower heads. We predict that species such as *Opius zacapuensis* Fischer, with head color pattern similar to the species described below from flower heads, will likewise be found to attack tephritids in this host plant microhabitat. Members of both the *godfrayi* and *pipitae* species groups are only known thus far from stem galling Tephritidae, suggesting that the members of these species groups may be specific to this plant niche. Our confidence in making such predictions is moderated to some extent by the new host record for *Doryctobracon anneae* Wharton, sp. n., feeding on a flower-infesting tephritid. All previously recorded hosts for the species of *Doryctobracon* Enderlein are frugivorous tephritids (Fischer 1977, Wharton and Marsh 1978). Except for the newly described species of *Doryctobracon*, the species described in the present work belong to species groups of *Opius* that are morphologically very distinct from the few known species of *Opius* that have been reared from fruit-infesting Tephritidae (Wharton 1997a, Wharton and Yoder 2012). Known parasitoids of leaf-mining tephritids also belong to completely different groups within the Opiinae, with New World species in both *Utetes* and *Eurytenes* Foerster (Wharton et al. 2012).

Nearly all of the host plants recorded here are from Asteraceae, the most important host plant family for non-frugivorous Tephritidae (Korneyev 1999, Norrbom 2010). There are only two exceptions. *Eutreta margaritata* Hendel, the tephritid host for *Opius dablus* Wharton, sp. n., was reared from *Penstemon* Schmidel, a new host plant genus and plant family (Plantaginaceae) for the Tephritidae. *Lippia substrigosa* Turcz., a member of the Verbenaceae, and host of *Opius baderae* Wharton, sp. n., is a new host plant record for *Eutreta xanthochaeta* Aldrich, a tephritid previously known from other species of Verbenaceae. Within the Asteraceae, new fly/host plant associations are *Eutreta christophe* (Bates) from stem galls of *Dahlia imperialis* Roezl and *Eutreta apicata* Hering from *Squamopappus skutchii* (S.F. Blake) R.K. Jansen, N.A. Harriman & Urbatsch and *Podachaenium eminens* (Lag.) Sch. Bip. (see the descriptions of *Opius godfrayi* Wharton, sp. n. and *Opius marshi* Wharton, sp. n., respectively, for further details).

Rates of parasitization per sample ranged from 9–87% (excluding the two samples that produced a few wasps but no flies). This relatively high rate lends support to the hypothesis put forth by Ward et al. (2013) that opiine parasitoids of flower and stem-infesting tephritids are primarily a tropical group, at least in the New World, since large samples of flowerheads from several sites in the U.S. have produced abundant flies but no opiines. Comparably large samples for stem-infesting tephritids from the Nearctic are unfortunately not available. Given the new host recorded below for the Nearctic *Opius peleus* Fischer, additional sampling is warranted to test the hypothesis that opiine parasitism of stem-inhabiting tephritids is similarly rare in the Nearctic.

The hosts of most of the species described below were collected in the fall at relatively high elevations. In most cases, both hosts and parasitoids overwintered and emerged the following year. Diapause in essentially tropical parasitoids is a relatively poorly studied phenomenon though it has been known since the seminal contributions to opiine biology by Pemberton and Willard (1918). Aluja et al. (1998) explored diapause in opiine parasitoids of neotropical fruit-infesting tephritids in some detail, and also noted that this phenomenon was essentially absent at lower elevations in contrast to samples taken above 1000 m. Denlinger (1986) provides a good though rather dated review of dormancy in tropical insects in general.

### Key to New World species reared from stem, leaf, and flower-infesting Tephritidae

This key includes all New World opiine parasitoids of non-frugivorous tephritids. The species of *Opius* (*Bellopius* Wharton), *Opius* (*Thiemanastrepha* Fischer), and *Utetes* are not treated here, but either have been (Wharton et al. 2012) or will be treated elsewhere. These three taxa are thus represented in the key only as genera or subgenera.

**Table:** 

1	Hind tibia with basal carina on posterior face (Fig. 5). Mesoscutum with depression (midpit) posterior-medially (Figs 15, 16, 85). Labrum exposed (as in Figs 57, 59, 64)	*Utetes* Foerster
–	Hind tibia normal, without basal carina. Mesoscutum in nearly all species without midpit. Labrum exposed or concealed	2
2 (1)	Occipital carina absent both dorsally and laterally (Figs 1, 85)	3
–	Occipital carina present and well-developed laterally, absent dorsally (Figs 2, 3)	6
3 (2)	Second submarginal cell short (Fig. 14)	*Doryctobracon anneae* Wharton
–	Second submarginal cell longer (Figs 17–20)	4
4 (3)	Mesoscutum without midpit (as in Figs 39, 53). Propodeum usually with median longitudinal carina, bifurcating posteriorly	5
–	Mesoscutum with long, deep, narrow midpit (Fig. 85). Propodeum without carinae	*Opius taramegillae* Wharton
5 (4)	Labrum exposed (as in Figs 59, 64). Fore wing m-cu entering second submarginal cell (as in Fig. 36)	*Opius (Thiemanastrepha)* spp.
–	Labrum concealed (as in Figs 37, 41, 47). Fore wing m-cu entering first submarginal cell (as in Fig. 90)	*Opius (Bellopius)* spp.
6 (2)	Occipital and hypostomal carina meeting ventrally distinctly above base of mandible, extending to base of mandible as a single, elevated flange (Fig. 3)	7
–	Occipital and hypostomal carina widely separated ventrally (Figs 2, 4)	9
7 (6)	Labrum narrowly exposed (Figs 55, 57). Mesopleuron extensively dark brown to black (Fig. 21)	*Opius godfrayi* Wharton
–	Labrum broadly exposed (Figs 59, 64). Mesopleuron mottled, with significant portions pale (Figs 24, 25)	8
8 (7)	Hind tibia pale. Notaulus barely indicated	*Opius nablus* Wharton
–	Hind tibia extensively infumate. Notaulus short but distinctly impressed	*Opius marshi* Wharton
9 (6)	Labrum completely concealed by clypeus when mandibles closed (Figs 37, 41, 47)	10
–	Labrum exposed when mandibles closed (Fig. 71)	16
10 (9)	Mesoscutum densely setose (Fig. 89). Fore wing m-cu distinctly antefurcal (Fig. 90); fore wing 2CUb arising from or posteriorad midpoint of distal margin of 1st subdiscal cell (Fig. 90)	*Opius yoderi* Wharton
–	Mesoscutum sparsely setose (Figs 49, 53). Fore wing m-cu postfurcal (Fig. 54); fore-wing 2CUb arising anteriorad midpoint of distal margin of 1st subdiscal cell	11
11 (10)	Head mostly dark above, pale below with nearly completely pale orbital ring (Figs 47–49)	12
–	Head completely dark above, white below, without orbital ring (Figs 41–46)	13
12 (11)	Female with metasomal terga 3–6 with broad, alternating dark and white transverse bands (Fig. 22); male terga 3–6 mostly dark. Body smaller, mesosoma 1.0–1.2 mm	*Opius gabriellae* Wharton
–	Female with metasomal terga pale (Figs 49, 50); male terga 3–6 mostly pale. Body larger, mesosoma 1.3–1.6 mm	*Opius danielsae* Wharton
13 (11)	Mesosoma extensively pale: mesopleuron completely pale, mesoscutum lacking large dark bands or spotches (Figs 18, 39)	*Opius baeblus* Wharton
–	Mesosoma darker: mesopleuron mostly dark, mesoscutum usually with dark bands and/or splotches (Figs 17, 19, 20)	14
14 (13)	Face with wedge of white coloration extending dorsally from malar region along inner margin of eye (Fig. 42). Lateral lobes of mesoscutum pale	*Opius dablus* Wharton
–	Face white below transverse line between dorsal-most portion of epistomal sulcus and ventral margin of eye, without additional wedge along inner margin of eye (Figs 37, 41). Lateral lobes of mesoscutum with dark bands in most specimens	15
15 (14)	Female with ovipositor 2.0 × longer than mesosoma. Mesosoma 1.2–1.5 mm long	*Opius baderae* Wharton
–	Female with ovipositor 2.25 × longer than mesosoma. Mesosoma 1.0 mm long	*Opius cablus* Wharton
16 (9)	Propodeum extensively sculptured: rugulose to rugose (Fig. 9)	17
–	Propodeum largely smooth and polished (Fig. 10)	19
17 (16)	T1 with dorsope absent (Fig. 9). Fore wing stigma wedge-shaped, distinctly narrowing distally	*Opius peleus* Fischer
–	T1 with dorsope present (Fig. 6). Fore wing stigma more or less parallel-sided	18
18 (17)	Mesoscutum densely setose, the setae obscuring base of notaulus (Fig. 7)	*Eurytenes norrbomi* Wharton
–	Mesoscutum less densely setose, setae never obscuring base of notaulus (Fig. 8)	*Eurytenes macrocerus* (Thomson)
19 (16)	Fore wing 2CUb arising below middle of distal margin of 1st subdiscal cell (Fig. 70). Mesoscutum with supramarginal carina distinct (Fig. 68)	*Opius nympha* Fischer
–	Fore wing 2CUb arising above middle of distal margin of 1st subdiscal cell (Figs 78, 82). Mesoscutum with supramarginal carina indistinct or absent (Fig. 76)	20
20 (19)	Mesosoma entirely black; head almost entirely black	*Opius stecki* Wharton
–	Mesoscutum and head extensively pale	*Opius pipitae* Wharton

**Figures 1–4.**
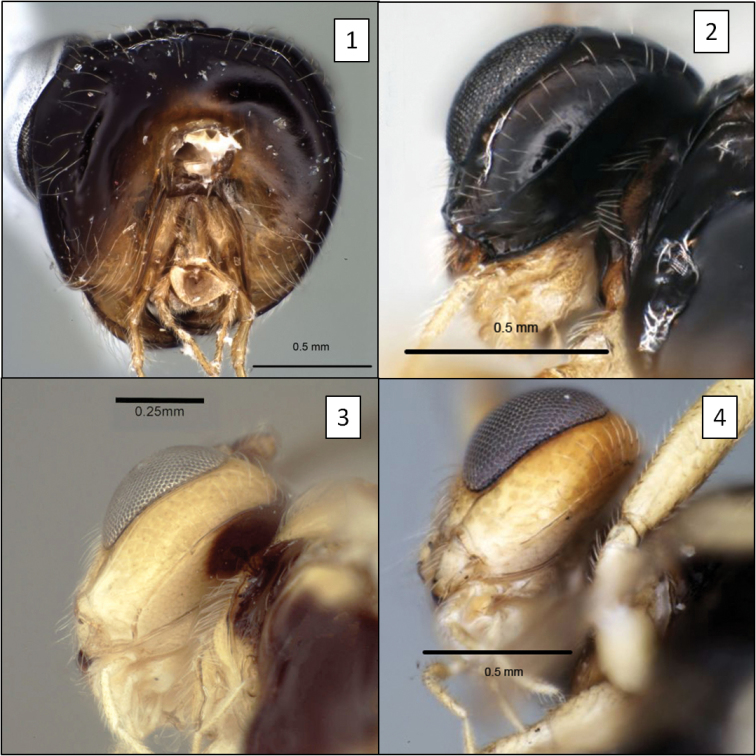
Opiinae spp., occipital and hypostomal carinae. **1** *Doryctobracon crawfordi* (Viereck) showing occipital carina completely absent **2** *Opius peleus* Fischer, showing the two carinae widely separated at mandible **3** *Opius godfrayi* Wharton, sp. n., with both carinae meeting well above base of mandible **4** *Opius nympha* Fischer, similar to *Opius peleus*.

**Figures 5–8.**
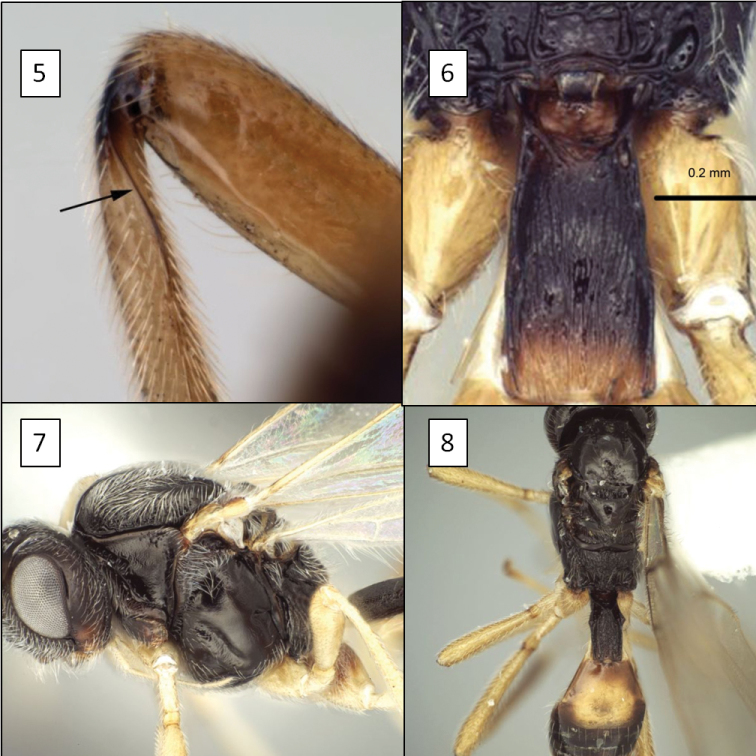
Opiinae spp. **5** *Utetes anastrephae* (Viereck) showing hind tibia with basal carina (arrow) **6** *Eurytenes maya* Wharton, T1 with dorsope **7** *Eurytenes norrbomi* Wharton, mesoscutum lateral **8** *Eurytenes macrocerus* (Thomson), dorsal habitus.

**Figures 9–12.**
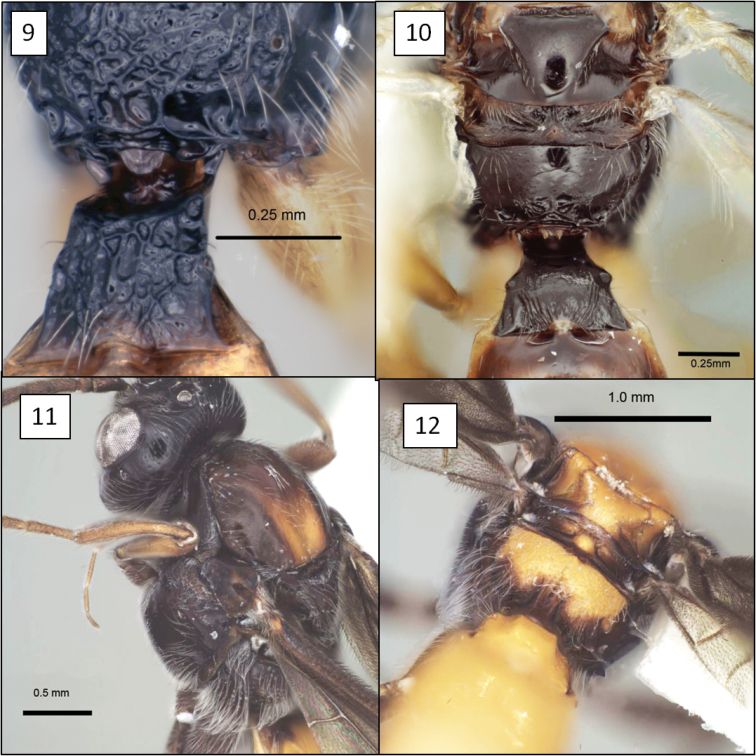
Opiinae spp. **9** *Opius peleus* Fischer,propodeum and T1 without dorsope **10** *Opius danielsae* Wharton, sp. n., propodeum **11** *Doryctobracon anneae* Wharton, sp. n., with dark mesosoma **12** *Doryctobracon anneae*, propodeum.

### Taxonomy

The species treated below are remarkably diverse, representing seven morphologically distinct groups within *Opius* s.l. and one additional species in the genus *Doryctobracon* Enderlein. *Doryctobracon* is treated first, followed by *Opius* s.l. Three species groups of *Opius* s.l. are formally described for the primary purpose of avoiding unnecessary repetition in species descriptions of the multiple species contained in each of these three groups. In these species groups, a narrow species concept has been employed, based heavily on host relationships when differences in hosts were supported by at least small differences in morphology.

Species and species group descriptions are in alphabetical order. Unfortunately, some of the species are known only from singletons, but are described here to emphasize the diversity of species attacking stem mining and flower infesting tephritids in the New World.

#### Doryctobracon

Enderlein

http://species-id.net/wiki/Doryctobracon

Doryctobracon Enderlein, 1920: 144. Type species: *Doryctobracon conjungens* Enderlein, 1920 [a junior subjective synonym of *Doryctobracon crawfordi* (Viereck, 1911)]. Original designation.
Parachasma Fischer 1967: 7. Type species: *Opius zeteki* Muesebeck, 1958. Original designation. Synonymized by Fischer (1977: 949).

##### Diagnosis.

Mandible without basal lobe ventrally. Labrum varying from almost completely concealed to partially but distinctly exposed ventrad margin of clypeus. Clypeus with ventral margin varying from weakly sinuate, nearly truncate, to strongly sinuate. Malar space distinct, malar sulcus absent. Occipital carina completely absent. First flagellomere equal to or slightly shorter than second, with dense patch of placoid sensilla laterally. Propleuron without oblique carina dorsad propleural flange. Pronotum lacking pronope but sometimes with small median pit adjacent posterior margin. Notauli usually distinctly impressed anteriorly, weak to nearly absent in some species. Supramarginal carina absent. Mesoscutal midpit weak to absent. Precoxal sulcus, sternaulus, and postpectal carina absent. Hind tibia dorsal-posteriorly without basal carina. Tegula overlapping and concealing most of basal wing sclerite. Fore wing stigma broad, distally discrete, with r1 arising near its midpoint; second submarginal cell short; m-cu nearly always (95%) antefurcal to interstitial with respect to 2RS, rarely weakly postfurcal. Hind wing RS absent, at least basally; m-cu long, nearly reaching wing margin, well-pigmented. T1 with deep laterope; dorsope absent. T2 and following terga without sculpture. Ovipositor long, always extending well beyond apex of metasoma.

**Remarks.** Members of this genus are native to the New World and are readily recognized by the combination of a short second submarginal cell (Fig. 14), complete absence of the occipital carina (Fig. 1), unsculptured notauli, and position of fore wing m-cu relative to 2RS. Species of *Doryctobracon* most closely resemble those species of *Diachasmimorpha* Viereck with reduced occipital carinae but differ primarily in the position of fore wing m-cu, the larger tegula, and the elevated posterior margin of the pronotum dorsally. Fischer (1977) and Wharton (1997a) provide redescriptions of *Doryctobracon*. Wharton and Marsh (1978), Fischer (1977) and Wharton and Yoder (2012) offer keys to species. There are two well-defined species groups based on differences in propodeal sculpture (Wharton 1997a). Hosts are known for nearly all described species, and all are parasitoids of larval Tephritidae, primarily of frugivorous species.

**Figures 13–16.**
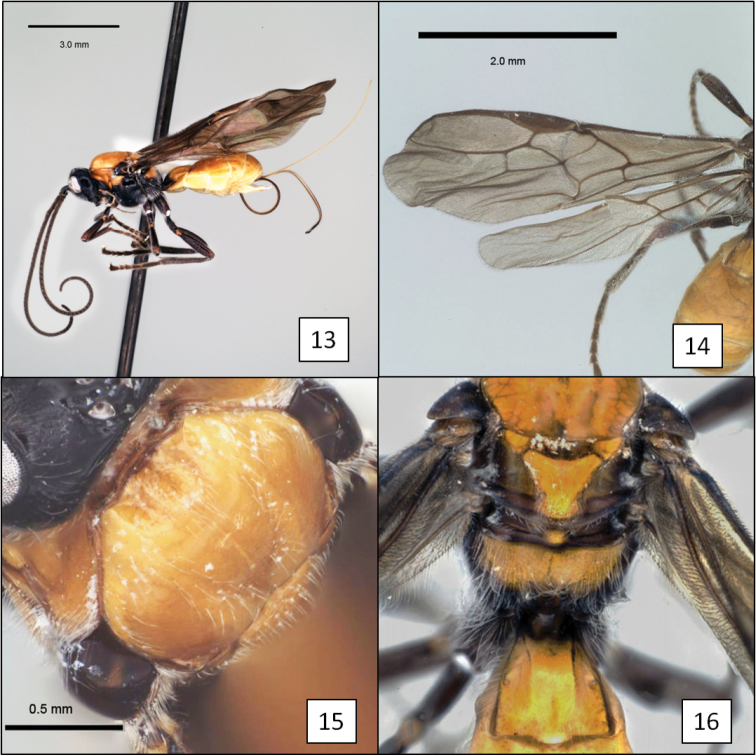
*Doryctobracon anneae* Wharton, sp. n. **13** habitus **14** wings **15** mesoscutum **16** T1.

#### Doryctobracon anneae

Wharton sp. n.

http://zoobank.org/E1E55966-B577-4071-8059-809CB4F6FDA0

http://species-id.net/wiki/Doryctobracon_anneae

Figs 11 –16

##### Type locality.

Mexico, Morelos, Lago de Zempoala.

##### Type material.

Holotype. Female (UNAM), first label, first line: MEXICO: Morelos second line: Lago de Zempoala third line: 23–25.ix.1991 fourth line: A. L. Norrbom, #57 Second label, first line: reared ex. capitulae second line: *Dahlia imperialis* third line: Roezl. (91M16) Third label, first line: reared ex puparium second line: *Gymnocarena mexicana* third line: (Tephritidae).

**Paratypes:** 3 males, 8 females, same data as holotype (USNM, TAMU). 1 male, 3 females, same data as holotype but reared ex. capitulae *Dahlia merckii* Lehm. (91M12A) (USNM, TAMU). 2 males, 5 females, 1 sex unknown (partly emerged from puparium), Morelos, Km 9–10 between Huitzilac and Lago de Zempoala, hollow on right, 22.ix.1991, A. Norrbom, reared ex capitulae *Dahlia merckii* (91M12), reared ex puparium *Gymnocarena mexicana* (USNM, TAMU). 3 males, 3 females, Michoacan, 2–4 km N. Angangueo, 4–5.ix.1991, A. L. Norrbom, reared ex. capitulae *Dahlia imperialis* (91M16B), reared ex. puparium *Gymnocarena mexicana* (USNM, TAMU, UNAM).

##### Description.

Eyes in dorsal view not or only slightly bulging beyond temples, temples not receding. Eye in lateral view 1.55–1.75 (male) and 1.7–1.9 (female) × longer than temple. Malar space large, greater than basal width of mandible, 0.45–0.6 × eye height. Clypeus sinuate, distinctly protruding as a lobe medially on ventral margin. Antenna with 41–47 flagellomeres; first flagellomere 0.8–0.9 × length of second, 1.5–1.75 × longer than wide; second flagellomere 1.75–2.0 × longer than wide. Mesosoma 1.3–1.4 × longer than high, 1.35–1.45 × higher than wide, 1.8–1.9 × longer than wide. Pronotum dorsally a broad, flat plate with weakly crenulate, shallow transverse groove near posterior margin, usually with small dimple-like depression dorsal-medially within groove; pronotum laterally with shallow, sinuate vertical groove, carinately margined on anterior side over dorsal 0.25; roughly elliptical area near middle of posterior-ventral margin delineated by very weakly crenulate groove. Notaulus virtually absent, represented primarily by a band of setae extending from anterior-lateral margin of mesoscutum to broad, shallow, median depression at posterior margin. Propodeum densely setose and punctate, with a pair of broadly rounded carinae extending anteriorly from median boss at posterior margin, carinae never extending to anterior margin, usually reaching midpoint; pleural carina often absent, sometimes weakly indicated on posterior 0.4–0.5. Metapleuron densely setose and punctate. Fore wing 2RS 1.3–1.6 × longer than 3RSa. T1 1.05–1.1 (male) and 1.15–1.3 (female) × longer than apical width, apex 1.9–2.3 (male) and 2.25–2.4 (female) × wider than base; T1 dorsal carinae parallel-sided, usually extending to level of spiracle as distinctly elevated ridges, then gradually weakening, not reaching posterior margin; spiracle posteriorad midpoint. Ovipositor 3.1–3.2 × and ovipositor sheath 2.6 × longer than mesosoma; ovipositor without subapical dorsal node. Head black; antenna, legs, ventral 0.6–0.4 of mesosoma, tegula, extreme base of T1 and ovipositor sheath dark brown, palps brown; mesosoma dorsally usually (90%) and metasoma entirely yellow-orange. Body length 3.8–6.0 mm; wing length 4.2–6.6 mm; mesosoma length 1.4–2.25 mm, with smallest male considerably smaller than smallest female.

##### Diagnosis.

This species is easily separated from the species of *Opius* s.l. treated below by the short second submarginal cell, with 3RSa much shorter than 2RS, and from all but *Opius taramegillae* by the complete absence of an occipital carina. The virtually absent notaulus separates *Doryctobracon anneae* from nearly all other species of *Doryctobracon*. The notaulus is also relatively poorly developed in *Doryctobracon homosoma* (Fischer), but the latter has an orange head and a bright yellow spot around the stigma on the otherwise infumate fore wing.

##### Biology.

All available specimens were reared from the tephritid *Gymnocarena mexicana* (Aczél) infesting flower heads of the asteraceans *Dahlia imperalis* and *Dahlia merkii* Lehm. Most of the host puparia from which the wasps emerged are stored in gelatin capsules on four separate pins. A few are associated with the individual wasps that produced them. Host records are detailed in Norrbom (2006: 223). Data on a few of the labels indicated that at least some of the parasitoids emerged from their hosts one year after the flower heads were collected. Flies in all four samples were heavily attacked by this opiine, with parasitism rates of 85.7, 80.0, 87.5, and 66.7% for collections 91M16, 91M12A, 91M12, and 91M16B, respectively.

The apex of the ovipositor is narrower in *Doryctobracon anneae* relative to species such as *Doryctobracon crawfordi*, which may indicate an earlier host stage attacked but may also be a reflection of the differences in host habitat (fruit vs flower head).

##### Etymology.

This species is named after Anne Wharton, deceased, wife of the senior author.

##### Remarks.

This new species differs substantially from the species of *Doryctobracon* known as parasitoids of fruit-infesting Tephritidae by the near absence of notauli. Nevertheless, the wing venation and shape of the head and clypeus clearly place this species within *Doryctobracon*. *Doryctobracon anneae* belongs to the *Doryctobracon crawfordi* species group characterized by the propodeal sculpture reduced to a pair of median carinae emanating from the posterior margin of the propodeum.

There is variation in the color pattern among the specimens available for study and although they were reared from two different host plants, specimens from *Dahlia imperialis* exhibited the maximum extent of variation. Ten percent of the specimens, representing two males and one female, all reared from *Dahlia imperalis*, have a dark mesoscutum while all others are completely pale. Two specimens, also from *Dahlia imperalis*, have the mesosoma ventrally much less extensively dark, with the tegula only partially brown.

#### *Opius* Wesmael s.l.

*Opius* s.l. is treated here in the sense of Wharton (1997a, b, 2006) and Wharton et al. (2012). It includes both *Phaedrotoma* and *Rhogadopsis* of Li et al. (2013), as noted above in the introduction.

Members of the *baderae*, *godfrayi*, and *pipitae* species groups are united by the shared characteristic of the anterior migration of the distal abscissa of the cubitus, which arises above the middle of the hind margin of the first subdiscal cell. The venation of the fore wing is similar in general among the species in these three species groups as is the nature of the notaulus (lacking, for example, a supramarginal carina). *Opius nympha*, *Opius peleus*, and *Opius yoderi* differ from members of these three species groups and from each other by sculpture patterns on the propodeum and metasomal tergites, shape of the mandible, and major differences in wing venation as detailed below under the species treatments. *Opius taramegillae* differs from all of these by the complete absence of an occipital carina and hence would be placed in *Bracanastrepha* in Fischer (1977).

#### Opius baderae species group

**Description.** Head: Occipital carina broadly absent middorsally, extending laterally from base of mandible to at least mid eye height, often to dorsal margin of eye in lateral view, widely separated from hypostomal carina ventrally. Malar space large, approximately as long as basal width of mandible. Clypeus tall, completely concealing labrum when mandibles closed; ventral margin in anterior view uniformly convex, thin but not impressed; flat in profile, with ventral margin often weakly protruding medially but never with horn or spine-like protrusions; epistomal sulcus narrow and deep throughout. Mandible narrowed apically, but not strongly so, apical teeth slightly twisted with ventral tooth smaller and more posteriorly positioned; dorsal margin nearly straight, weakly deflected; base of mandible not expanded ventrally to form an additional tooth or lobe. Maxillary palp longer than head height, usually distinctly so. Antenna longer than body, with at least 30 flagellomeres; first flagellomere distinctly longer than second. Face, gena, and frons largely smooth, never strongly sculptured.

Mesosoma: Pronotum dorsally a flat, narrow band, enlarged pronope absent, though sometimes with shallow median dimple; pronotum laterally with narrow, polished, unsculptured band bordering anterior margin separated along its full length from large, triangular, polished, unsculptured posterior portion by distinct groove, groove often sculptured, at least in part, more rarely carinate along anterior margin. Propleuron without oblique carina or groove dorsad propleural flange. Mesoscutum elevated anteriorly relative to pronotum, with distinct, nearly vertical anterior declivity; largely bare, with decumbent white setae densely covering lateral portions of anterior declivity up to base of notaular pit, more sparsely setose medially on declivity and along lateral margin between notaulus and tegula, row of shorter, decumbent setae scattered in decreasing density along traces of notaulus to posterior margin; without midpit posteriorly; notaulus comma-shaped: a short, curved groove extending posteriorly from a rounded pit, deep anteriorly, increasing shallow posteriorly, not extending to anterior margin nor posteriorly to level of tegula, not margined anteriorly by carinae; supramarginal carina absent parallel to lateral margin of mesoscutum between notaulus and tegula. Scuto-scutellar sulcus densely crenulate, very narrow, at least 8 × wider than mid length. Scutellum, parascutellar field, and flat band along posterior margin of mesothorax unsculptured; scutellum continuous with posterior band, not separated by depression or sculpture. Mesopleuron with subalar ridge rounded, not carinately margined ventrally, depression along ventral side smooth and unsculptured throughout; true sternaulus absent; precoxal sulcus very weakly impressed, rarely completely absent, never sculptured, short, not extending to anterior or posterior margins; mesopleural sulcus along posterior margin ventrad mesopleural fovea without obvious sculpture. Midventral longitudinal sulcus of mesothorax finely but distinctly crenulate. Metapleuron unsculptured medially; median pit adjacent anterior margin and dorsal pit at posterior margin both relatively small, not directly connected medially by a sulcus; ventral margin without well-developed spine anteriorly, at most with ventral carina weakly, unobtrusively expanded anteriorly. Propodeal spiracle closer to anterior than posterior margin; pleural sulcus usually distinct from spiracle to posterior margin; propodeum largely unsculptured, without median carina or median areola, usually with pair of short lateral-median longitudinal carinae apically. Metasomal and hind coxal cavities confluent: not separated by sclerotized bridge.

Legs and wings: Hind tibia without basal carina. Wings hyaline. Fore wing stigma narrow, tapered, with r arising basad its midpoint and separated from extreme base of stigma by at least its own length; 1RS short, 1M 6–10× longer than 1RS; 2RS present, sinuate, often strongly so, not thickened medially, 3RSa at least 1.6× longer than 2RS, 3RSb evenly bowed, extending to apex of wing or nearly so, not foreshortened; 2nd submarginal cell narrowing distally, height at r 1.4–1.65 × height at r-m; m-cu postfurcal; 2CUa distinctly shorter than 2cu-a, 2CUb thus arising anteriorad middle of distal margin of 1st subdiscal cell; 1st subdiscal cell slightly expanded distally; shortest distance between anal vein and ventral wing margin equal to 1–2× width of anal vein. Hind wing with 3 hamuli; RS largely spectral, sometimes weakly pigmented basally, much weaker than M; M distinct, usually tubular over at least basal 0.3, sometimes nebulous basally; m-cu completely absent.

Metasoma: S1 short, extending less than half distance to T1 spiracle. T1 distinctly and evenly broadening apically, never parallel-sided; distinct median basal depression not delimited posterior-medially by carina or other sculpture, delimited laterally by elevated basal portion of dorsal carina, dorsal carina weaker posteriorly, often becoming obsolescent; lateral carina well developed basally, meeting dorsal carina dorsad small, deep, basal laterope; dorsope absent. T2 and remaining terga unsculptured. Hypopygium large, broadly triangular, sharply pointed apically. Ovipositor with small dorsal node near apex.

**Diagnosis.** Members of the *Opius baderae* species group will key to *Opius (Opius)* in the subgeneric keys of Fischer (1972, 1977, 1999) because of the completely concealed labrum, unsculptured precoxal sulcus, and absence of a midpit on the mesoscutum. They differ from the type species of *Opius* (i.e., *Opius* s.s.) in lacking a basal lobe ventrally on the mandible, and thus would key to *Phaedrotoma* in the classification of van Achterberg and Salvo (1997) and Li et al. (2013) and the key to genera in Fischer (1999).

**Remarks.** *Opius zacapuensis* Fischer, from Michoacan, Mexico is also a member of this species group. It is known only from the male holotype and though consequently presenting some difficulties for comparison with the species described below it is nevertheless darker than any of these. *Opius aldrichi* Fischer represents a moderately large group of species that closely resemble members of the *baderae* species group but have the propodeum heavily and extensively sculptured. The propodeum is smooth in members of the *baderae* species group, with only a trace of weak sculpturing posteriorly. In addition to *Opius zacapuensis*, the following newly described species are included in the *baderae* species group: *Opius baderae*, *Opius baeblus*, *Opius cablus*, *Opius dablus*, *Opius danielsae*, and *Opius gabriellae*. The known species in this group range from Durango, Mexico to Guatemala. A species from eastern U.S., *Opius townesi* Fischer, was placed by Fischer (1977) in *Opius (Opius)*, and is somewhat similar to members of the *baderae* species group but has the labrum narrowly exposed and is therefore treated below under the *Opius pipitae* species group.

There appears to be some interspecific variation in the shape of the fore wing stigma, most notably in the relative width and how abruptly the stigma narrows apically. Unfortunately, the stigma is variably folded and curled in all of the specimens, making comparisons among species difficult. This applies to virtually all of the species described below, thus affecting comparisons within the *baderae* species group as well as across species groups.

#### Opius baderae

Wharton sp. n.

http://zoobank.org/23B1AF22-5D62-4672-9DB0-D837F7BC9FB8

http://species-id.net/wiki/Opius_baderae

Figs 17 , 33–36

##### Type locality.

Mexico, Chiapas, Chiquihuites, 15°05'N, 92°06'W.

##### Type material.

Holotype. Female (UNAM), first label, first line: MEXICO: Chiapas second line: Chiquihuites, -15°05'N third line: 92°06'W, Union Juarez, Second label, first line: S slope Volcan Tacaná second line: 1800–2000m, 31.x.1993 third line: A.L. Norrbom & C. Estrada Third label, first line: reared ex. stem galls second line: *Lippia substrigosa* third line: Turcz (93M7) Fourth label, first line: reared ex. puparium second line: *Eutreta xanthochaeta* third line: (Tephritidae).

**Paratypes:** 2 males, 1 female, same data as holotype (USNM, TAMU).

##### Other specimens examined

**(not paratypes):** 1 female, Mexico, Durango, 10 miles W El Salto, 9000 ft, 5.vii.1964, W.R.M. Mason (CNC); 1 male, Guatemala, Quiche, 2 km S Chichicastenango, on Rio Tesoro, 11.ix.1987, M. Sharkey (CNC).

##### Description.

Eyes in dorsal view not or only slightly bulging beyond temples, temples not receding. Clypeus 1.5–1.6 × wider than high, weakly punctate throughout; completely concealing labrum when mandible closed, ventral margin of clypeus evenly convex, slightly overlapping dorsal margin of mandible when mandible closed. Antenna with 39–43 (male) and 44–45 (female) flagellomeres. Malar sulcus weak, deeper adjacent eye, becoming shallower towards mandible. Mesosoma 1.25–1.3 (male) and 1.2 (female) × longer than high. Pronotum laterally with vertical groove varying from almost completely smooth and unsculptured to crenulate throughout, margined anteriorly by carina dorsally and ventrally in some specimens, distinct carina absent in others. Propodeum largely unsculptured, with a few weak carinulae along posterior margin, especially medially. Fore wing 3RSa 1.75–1.95 × longer than sinuate 2RS; (RS+M)a very weakly sinuate. T1 2.2–2.35 × wider at apex than at base, 0.95–1.1 × as long as apical width; finely striate over apical 0.7, smooth basally; dorsal carina extending to apical margin of T1 but low and weakly differentiated over posterior 0.5–0.7, not strongly elevated basally. Ovipositor (total length) 2.0 × longer than mesosoma; ovipositor sheath 1.5–1.6 × longer than mesosoma. Head entirely black to dark red-brown above, usually with small, light brown spot between base of antenna and eye, entirely white below horizontal line extending laterally from dorsal margin of clypeus through ventral margin of eye to occipital carina, base of mandible and all remaining mouthparts also white. Mesosoma black except propleuron pale to dark yellow, tegula and basal wing sclerite pale yellow, axillae and lateral 0.2–0.3 of metanotum yellow to dark yellow, and mesoscutum variegated: yellow with dark brown to black median band over anterior 0.75 and a dark blotch covering most of lateral lobe on each side. Metasomal terga dark brown to black; T3–T7 with narrow hyaline margin posteriorly, T7 band broader in female; T4–T6 also with median white band anteriorly. Fore and mid tibiae and all femora white, hind femur usually with pale brown subapical spot; hind tibia dark brown over basal 0.2, brown posteriorly over at least apical 0.5, otherwise variegated: usually paler subbasally, dorsally, and anteriorly, varying from whitish or dark yellow to brown. Body length 3.2–3.8 mm; wing length 3.8–4.85 mm; mesosoma length 1.2–1.5 mm. Otherwise having all the characteristics described above for the *baderae* species group.

**Figures 17–20.**
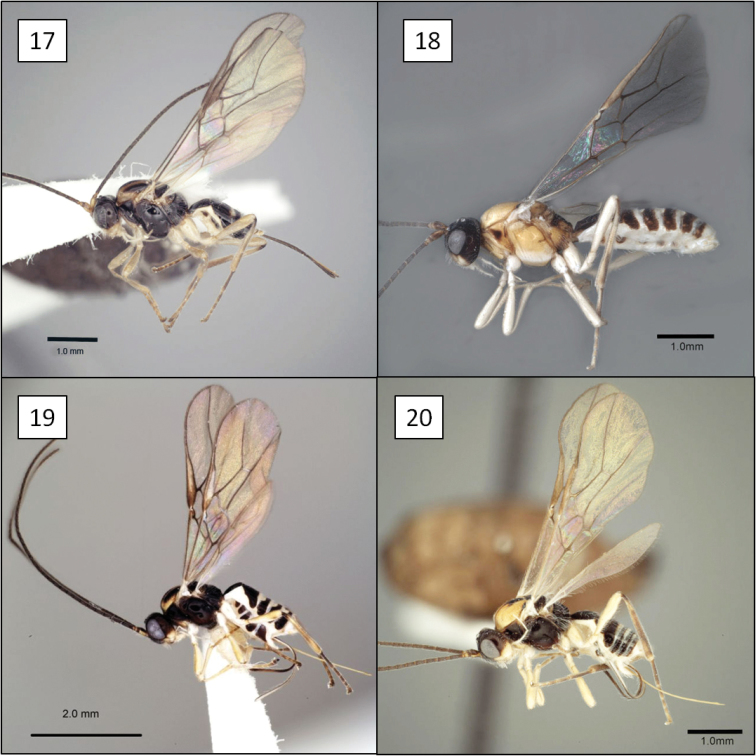
*Opius* spp., habitus. **17** *Opius baderae* Wharton, sp. n. **18** *Opius baeblus* Wharton, sp. n. **19** *Opius cablus* Wharton, sp. n. **20** *Opius dablus* Wharton, sp. n.

##### Diagnosis.

This species is nearly identical to *Opius cablus* sp. n., described below, but the ovipositor is slightly shorter in relation to body size. *Opius baderae* attacks a larger host tephritid and is consequently distinctly larger than *Opius cablus*.

##### Biology.

*Lippia substrigosa* is a new host plant record for *Eutreta xanthochaeta*. The fly is best known as the lantana gall fly for its use in Hawaii and Australia, where it was purposefully introduced early in the 1900s as a biological control agent for the introduced weed *Lantana camara* L. Both *Lippia substrigosa* and *Lantana camara* are members of the Verbenaceae. For this sample of stem galls, the rate of parasitism was 29.4%.

##### Etymology.

This species is named for Amy Bader, who assisted with preliminary work on this species group.

##### Remarks.

One of the three males is considerably smaller than the other four specimens, with wing length 0.4 mm less than the next largest specimen, but otherwise matches the remainder of the reared series. The two non-paratypes vary slightly in the color of the mesoscutum and since they are also unassociated with hosts, they have been excluded from the paratype series.

For all specimens, the apparent color pattern on the metasoma varies with preservation. The anterior median white patches are not fully visible when the terga are in their normally retracted position. Similarly, the hyaline posterior margins are not readily visible in normally retracted position because they lie over the dark part of the tergite.

#### Opius baeblus

Wharton sp. n.

http://zoobank.org/91420AA7-9AFF-4BD5-B183-8405BFD5435C

http://species-id.net/wiki/Opius_baeblus

Figs 18 , 37–40

##### Type locality.

Mexico, Morelos, Route 142, Km 48–50, 5 km N El Vigia.

##### Type material.

Holotype. Male (UNAM), first label, first line: MEXICO: Morelos, Rt. second line: 142, Km 48–50, 5 km N third line: El Vigia, 28.ix–1.x.1991 fourth line: A. L. Norrbom # 51 Second label, first line: reared ex. spherical stem second line: gall, *Ageratina mairetiana* third line: (DC) K & R. (91M27) Third label, first line: host is Eutreta sp. second line: apicata Hering or n. sp. third line Tephritidae) Fourth label, first line: reared ex. pupae second line: ex. galls 91M27 third line: emg. 1.vii.1992.

##### Description.

*Male*. Eyes in dorsal view slightly bulging beyond temples, temples weakly receding. Clypeus 1.6 × wider than high, weakly rugulose dorsal-medially adjacent epistomal sulcus, weakly punctate elsewhere; completely concealing labrum when mandible closed, ventral margin of clypeus evenly convex, slightly overlapping dorsal margin of mandible when mandible closed. Antennae broken, right antenna with 36 flagellomeres remaining. Malar sulcus broad, weak, deeper adjacent eye, becoming shallower towards mandible. Mesosoma 1.35 × longer than high. Pronotum laterally with complete vertical carina, carina weaker medially, distinctly crenulate along posterior margin of carina in ventral 0.3, smooth medially, weakly crenulate dorsally. Propodeum largely unsculptured, with a few weak carinulae along posterior margin. Fore wing 3RSa 1.75 × longer than strongly sinuate 2RS; (RS+M)a very weakly sinuate. T1 2.1 × wider at apex than at base, 0.9 × as long as apical width; strigose over apical 0.7, smooth basally; dorsal carina arising at 45 degree angle alongside basal depression, absent over apical 0.7. Head entirely black to dark red-brown above, ventral 0.7 of clypeus, nearly entire malar space, base of mandible, and all remaining mouthparts white. Mesosoma almost completely yellow-orange except pronotum laterally with dark spot medially and propodeum dark medially and anteriorly. Metasomal terga dark brown to black; T3–T6 with narrow white or hyaline margin posteriorly, band broader on T7; T4–T6 also with narrow median white band anteriorly. Fore and mid tibiae and all femora white; hind tibia brown dorsally and posteriorly over apical 0.3, variegated brown over basal 0.25, white medially. Body length 5.15 mm; wing length 5.1 mm; mesosoma length 1.7 mm. Otherwise having all the characteristics described above for the *baderae* species group.

##### Diagnosis.

This species is most readily recognized by the pale mesosoma (Fig. 18). All other members of the *baderae* species group treated here have the mesosoma extensively dark, at least laterally (Figs 17, 19, 20). *Opius baeblus* is most similar to *Opius baderae* based on the color pattern of the head, most notably the gena, face and clypeus, and differs primarily in body color, larger body size, and in having the anal vein of the fore wing more distant from the wing margin.

##### Biology.

Nine tephritids emerged from these stem galls within two months of their collection. Seven of these tephritids are *Eutreta apicata* and two represent an undescribed species of *Eutreta* Loew. The wasp overwintered in the laboratory and emerged the following summer. For this sample, the rate of parasitism on *Eutreta* spp. by opiine braconids was 10%. The host plant, *Ageratina mairetiana* (DC.) R.M. King & H. Rob., is a member of the Asteraceae.

##### Etymology.

The species name is an arbitrary combination of letters.

##### Remarks.

Despite the fact that this species is known from a single male, it is described here to emphasize the diversity of color patterns and host relationships of the members of this distinctive species group of tephritid parasitoids. 3RSa is longer in the right wing than in the left wing.

#### Opius cablus

Wharton sp. n.

http://zoobank.org/6F9E8C03-4376-4A68-B9C0-ACD87F95E8FE

http://species-id.net/wiki/Opius_cablus

Figs 19 , 41 , 43 , 45

##### Type locality.

Guatemala, Sacatepequez, Volcan de Agua, trail from Ciudad Viejo.

##### Type material.

Holotype. Female (USNM), first label, first line: Guatemala: Sacatepequez: second line: Volcan de Agua, trail third line: from Ciudad Viejo, fourth line: 19. X. 1990, A.L.Norrbom Second label, first line: reared ex. stem of second line: undetermined plant (90G13) third line: probably ex. puparium fourth line: of Tephritidae sp., possibly fifth line: Trupanea sp.

**Paratype:** 1 female, same data as holotype (TAMU).

##### Description.

*Female*. Eyes in dorsal view slightly bulging beyond temples, temples weakly receding. Clypeus 1.26–1.4 × wider than high, very weakly punctate throughout; completely concealing labrum when mandible closed, ventral margin of clypeus evenly convex, slightly overlapping dorsal margin of mandible when mandible closed. Antenna with 38 and 42 flagellomeres. Malar sulcus distinctly impressed throughout. Mesosoma 1.2 × longer than high. Pronotum laterally with vertical groove weakly crenulate dorsally, distinctly crenulate ventrally, weakly sculptured medially. Propodeum mostly unsculptured, with small weakly rugulose patch posterior-medially. Fore wing 3RSa 1.85–1.95 × longer than sinuate to strongly sinuate 2RS; (RS+M)a weakly sinuate, nearly straight. T1 2.25–2.35 × wider at apex than at base, 1.1 × longer than apical width; smooth, unsculptured basally, striate to finely strigose over apical 0.5; dorsal carina distinct basally, extending to apex but weaker and largely obscured by sculpture posteriorly. Ovipositor (total length) 2.25 × longer than mesosoma; ovipositor sheath 1.8–1.85 × longer than mesosoma. Head color as in *Opius baderae*. Mesosoma black to dark red-brown except propleuron almost completely light brown, paler ventrally; tegula and basal wing sclerite pale yellow to white; mesopleuron with pale brown to brown band extending between fore and mid coxae; axillae and lateral 0.2–0.3 of metanotum dark yellow to yellow-orange; mesoscutum variegated as in *Opius baderae*, with 3 black bands on yellow-orange background. T1 black, T2–T6 dark reddish brown to black, T3–T6 with narrow hyaline margin posteriorly; T4–T6 also with median white band anteriorly. Fore and mid tibiae and all femora pale yellow, hind femur with pale brown subapical spot; hind tibia mostly yellow, dark brown over basal 0.2, with some weak infumation apically on posterior face. Body length 2.7–3.1 mm; wing length 3.3–3.6 mm; mesosoma length 1.0 mm. Otherwise having all the characteristics described above for the *baderae* species group.

##### Diagnosis.

This species is nearly identical to the larger-bodied *Opius baderae*, but there are slight differences in the color of the propleuron and the ovipositor is longer relative to the body length.

##### Biology.

The tephritid puparia from which the holotype and paratype emerged are mounted with each of the separately point-mounted specimens. The puparia are distinctly different from the puparia of the *Eutreta xanthochaeta* that yielded the type series of *Opius baderae*. They are smaller, black, and consistent with known species of *Trupanea*. No flies emerged from this sample of stem galls, but dissection of an unemerged puparium revealed remains of a tephritid that was probably a species of *Trupanea*. The plant had no reproductive structures and could not be identified.

##### Etymology.

The species name is an arbitrary combination of letters.

##### Remarks.

This species is known only from the two females reared from a tephritid host infesting stems of an unknown plant.

#### Opius dablus

Wharton sp. n.

http://zoobank.org/7D750D6A-013E-4680-A5C4-22337DF3E620

http://species-id.net/wiki/Opius_dablus

Figs 20 , 42 , 44 , 45 , 46

##### Type locality.

Mexico, Morelos, 5 km N El Vigia.

##### Type material.

Holotype. Female (UNAM), first label, first line: MEXICO: Morelos, Rt. second line: 142, Km 48–50, 5 km N third line: El Vigia, 28.ix.–1.x.1991 fourth line: A. L. Norrbom, #49 Second label, first line: reared ex. *Eutreta* second line: *margaritata* ex. stem gall third line: on *Penstemon kunthii* fourth line: C. Don. (91M13A) Third label, first line: reared ex. puparium second line: ex. gall 91M13A third line: emg. 30.v.1992.

##### Description.

*Female*. Eyes in dorsal view slightly bulging beyond temples, temples weakly receding. Clypeus 1.65 × wider than high, weakly punctate throughout; completely concealing labrum when mandible closed, ventral margin of clypeus evenly convex, slightly overlapping dorsal margin of mandible when mandible closed. Antenna with 40 flagellomeres. Malar sulcus weak, barely indicated near eye margin. Mesosoma 1.3 × longer than high. Pronotum laterally with vertical groove weakly crenulate dorsally, more distinctly crenulate ventrally, otherwise smooth and unsculptured. Propodeum unsculptured, with a few weak carinulae along posterior margin. Fore wing 3RSa 1.6 × longer than strongly sinuate 2RS; (RS+M)a straight. T1 2.35 × wider at apex than at base, 0.95 × as long as apical width; smooth, unsculptured; dorsal carina low not distinctly elevated basally, absent over apical 0.7. Ovipositor (total length) approximately 2.0 × longer than mesosoma; ovipositor sheath approximately 1.5 × longer than mesosoma. Head entirely black to dark red-brown above, entirely white below horizontal line extending from dorsal margin of clypeus through ventral margin of eye to occipital carina, with triangular wedge extending dorsally above this line between clypeus and eye; base of mandible and all remaining mouthparts also white. Mesosoma black to dark red-brown except propleuron, tegula and basal wing sclerite pale white; axilla and most of mesoscutum orange, with very narrow median black stripe ending posteriorly in large shield-shaped black spot. T1 black, T2–T6 dark reddish brown medially, T3–T6 with narrow hyaline margin posteriorly; T4–T6 also with median white band anteriorly. Fore and mid tibiae and all femora pale yellow; hind tibia mostly brown, darker brown over basal 0.2, with yellow band on middle 0.4 dorsally and dorsal-posteriorly, variegated anteriorly. Body length 3.0 mm; wing length 4.0 mm; mesosoma length 1.3 mm. Otherwise having all the characteristics described above for the *baderae* species group.

##### Diagnosis.

This species most closely resembles *Opius baderae*, *Opius baeblus*, and *Opius cablus* based on the color pattern of the head (dark above, white below) relative to the other members of this species group described here. In *Opius dablus*, there is a wedge of white that extends more dorsally along the inner eye margin than in the other three species, the hind tibia tends to be a little more evenly infumate, and T1 is not as heavily sculptured. *Opius baeblus* is larger and the mesosoma is more extensively pale than in the other three species, and in *Opius dablus* the lateral lobes of the mesoscutum are orange but mostly dark brown to black in *Opius baderae* and *Opius cablus*. The ovipositors of *Opius dablus* and *Opius baderae* are similar in length and shorter than in *Opius cablus*.

##### Biology.

Ten specimens of *Eutreta margaritata* were reared from the same collection of stem galls on *Penstemon kunthii* G. Don that produced the holotype, resulting in a parasitism rate of 9%. This is a new host plant record for this tephritid and the first record for any tephritid from *Penstemon* and the Plantaginaceae.

##### Etymology.

The species name is an arbitrary combination of letters.

##### Remarks.

This species is thus far known only from the female holotype. Five female specimens collected with sweep net in Guerrero and Oaxaca (TAMU) are nearly identical to this species. Although varying slightly in pattern, they all have the wedge of white color extending along the eye margin dorsally from the lateral margin of the clypeus and an orange mesoscutum with a dark median blotch. All of these, however, differ in having a slightly shorter ovipositor and sheath and thus are hypothesized to represent a separate but closely related species.

The fore wing 1RS is longer in this species than in others of this species group, at the lower end of the range for the 1M/1RS ratio given in the species group diagnosis. In other species in this group, the ratio is near the upper end of the range. Similarly, fore wing m-cu is more strongly postfurcal in *Opius dablus*, resulting in a lower 3RSa/2RS ratio than in most other members of this species group.

#### Opius danielsae

Wharton sp. n.

http://zoobank.org/2D3D0B1B-EF0E-4678-BFFD-A6B8DBD849B6

http://species-id.net/wiki/Opius_danielsae

**Figures 21–24.**
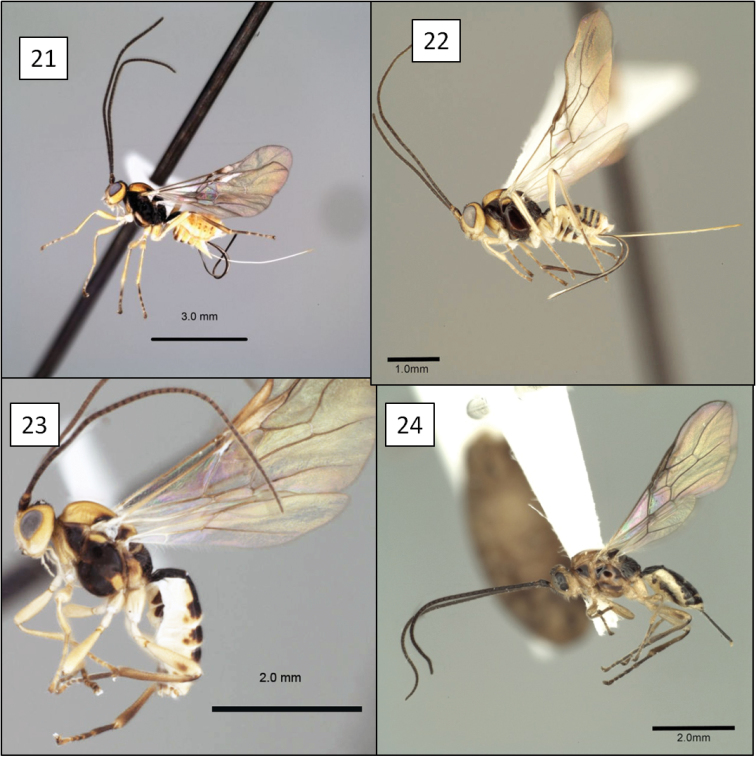
*Opius* spp., habitus. **21** *Opius danielsae* Wharton, sp. n. **22** *Opius gabriellae* Wharton, sp. n. **23** *Opius godfrayi* Wharton, sp. n. **24** *Opius marshi* Wharton, sp. n.

Figs 10 , 21 , 47–50

##### Type locality.

Mexico, Morelos, Lago de Zempoala.

##### Type material.

Holotype. Female (UNAM), first label, first line: MEXICO: Morelos second line: Lago de Zempoala third line: 23–25.ix.1991 fourth line: A. L. Norrbom Second label, first line: reared ex capitulum second line: of Dahlia imperialis third line: Roezl (91M16) Third label, first line: reared ex puparium second line: Laksyetsa trinotata third line: (Tephritidae) emer. fourth line: viii.1992.

**Paratypes:** 1 male, same data as holotype (USNM). 2 females, 1 male, 1? (still within host puparium), same data as holotype but without emergence date (TAMU, USNM). 1 male, same data as holotype but emerged 21.v.1992 (USNM). 1 female, same data as holotype but emerged vi.1992 (TAMU).

##### Description.

Temples in dorsal view bulging beyond eyes, not receding. Clypeus 1.65–1.75 × wider than high, distinctly punctate throughout; completely concealing labrum when mandible closed, ventral margin of clypeus evenly convex, slightly overlapping dorsal margin of mandible when mandible closed. Antenna with 39–42 (male) and 37–41 (female) flagellomeres. Malar sulcus narrow, shallow, distinct throughout. Mesosoma 1.25–1.3 × longer than high. Pronotum laterally with vertical groove usually weakly crenulate dorsally, distinctly crenulate ventrally, varying from smooth to weakly wrinkled medially, not margined anteriorly by carina. Propodeum mostly unsculptured, with small weakly rugulose patch posterior-medially. Fore wing 3RSa 1.7–1.9 × longer than sinuate 2RS; (RS+M)a very weakly sinuate, nearly straight. T1 2.0–2.15 (male) and 2.2–2.35 (female) × wider at apex than at base, 0.9–1.15 × as long as apical width; smooth, unsculptured basally, variously striate to strigose over apical 0.7: often weaker medially, sometimes mostly smooth; dorsal carina low, not distinctly elevated basally, weakening to absent or nearly so over apical 0.6. Ovipositor (total length) 3.1–3.2 × longer than mesosoma; ovipositor sheath 2.4–2.6 × longer than mesosoma. Head mostly black above, including at least dorsal 0.5 of occiput, dark color extending between and below antennae to cover middle of face with median dark brown spot, the spot slightly larger in female than male, usually extending narrowly to epistomal sulcus; remainder of face, orbit dorsally, lower occiput, and almost entire gena yellow fading to white on lower gena and malar region; orbital ring interrupted above antennal torulus by narrow black band extending laterally from frons; clypeus, mandible except dark apical teeth, and remaining mouthparts white to very pale yellow. Mesosoma black to dark red-brown except as follows: propleuron dark brown to variously infumate dorsally, white to pale yellow ventrally in female, pale throughout in male; tegula and basal wing sclerite pale white; axilla and most of mesoscutum orange with broad, median black band over anterior 0.6–0.7, band faded to dark orange in one specimen, anterior part of black band sometimes absent on anterior declivity, small black spot also present along lateral margin between posterior end of tegula and axilla; metanotum usually with margins at least partly yellow-brown; pleuron on each side between fore and mesocoxal cavities variably marked with orange. T1 black, T2 and anterior portion of T3 usually reddish brown with narrow yellow lateral margins, two specimens with T2 and T3 mostly or entirely yellow; T4–T7 and T3 posteriorly yellow with narrow hyaline margin posteriorly, rarely with narrow, dark brown transverse bands. Fore and mid tibiae and all femora pale yellow; hind tibia varying from almost completely brown to mostly yellow with at least basal 0.2 and apical 0.4 posteriorly brown, usually darker posteriorly than anteriorly. Body length 3.4–4.2 mm; wing length 4.5–4.8 mm (male), 4.25–4.45 mm (female); mesosoma length 1.55–1.6 mm (male), 1.3–1.5 mm (female). Otherwise having all the characteristics described above for the *baderae* species group.

##### Diagnosis.

This species is very similar to the distinctly darker *Opius zacapuensis* from Michoacan and the smaller-bodied *Opius gabriellae* described below. In all three of these species, the head is distinctively patterned, with frons, vertex, and upper occiput dark, face with a median infumate spot, remainder pale, including a pale orbital ring interrupted by a dark bar extending from mid frons to eye. Females and most males of *Opius danielsae* lack dark transverse bars on the metasomal segments, unlike individuals of the other two species. The lateral mesoscutal lobes are dark brown to black in *Opius zacapuensis* but orange in *Opius danielsae* and *Opius gabriellae*.

##### Biology.

Data on the host fly and host plant (including images of the latter) are published in Norrbom et al. (2010), where *Laksyetsa* Foote is treated as a junior subjective synonym of *Paracantha* Coquillett. The host for *Opius danielsae* is therefore *Paracantha trinotata* (Foote). Parasitism of *Paracantha trinotata* by *Opius danielsae* was 17.9% for this sample. Several of the *Paracantha* puparia from which these wasps emerged are card mounted on separate pins. The host plant, *Dahlia imperialis*, is a member of the Asteraceae.

Two distinctly different opiines were reared from this sample of *Dahlia imperialis* flower heads, with *Doryctobracon anneae* reared only from *Gymnocarena mexicana* and *Opius danielsae* reared only from *Paracantha trinotata*. The puparia of the two tephritids are distinctly different in color and texture, allowing reliable segregation prior to emergence of flies and wasps. One sample of the same plant species from the same general locality but two years earlier yielded three specimens of a third species of Opiinae, but without specific host associations. This wasp is described below as *Opius yoderi*.

##### Etymology.

This species is named for Sophia Daniels, without whose inspiration this work could not have been completed.

##### Remarks.

The seven specimens reared from this sample were fairly similar in color pattern, providing a basis for assessing inter- vs intraspecific patterns for the opiines described here. Females from this sample were slightly smaller than males, with T1 also broader apically than in males. The middle of the face was noticeably bulging in some specimens and barely so in others, independent of sex.

#### Opius gabriellae

Wharton sp. n.

http://zoobank.org/C9E6D1A1-29E1-44D6-BE52-9D231016EF13

http://species-id.net/wiki/Opius_gabriellae

Figs 22 , 51–54

##### Type locality.

Mexico, Mexico, 6 km West Lago de Zempoala.

##### Type material.

Holotype. Female (UNAM), first label, first line: MEXICO: Edo. de second line: Mexico third line: Rt. 890, Km 9 area fourth line: 6km W Lago de Zempoala fifth line: 2–x–1991 A. Norrbom Second label, first line: reared ex capitulum second line: Senecio iodanthus third line: Greenm. 91(M)33 fourth line: Probably ex. puparium fifth line: Paroxyna (Tephitidae).

**Paratypes:** 1 male, 1 female, same data as holotype (TAMU, USNM).

##### Other specimen examined

**(not paratype):** 1 female, Mexico: Morelos, Rt. 142, Km 48–50, 5 km N El Vigia, 28.ix–1.x.1991, A. L. Norrbom, reared ex. capitulum *Montanoa frutescens* Mairet ex. DC (91M5B) (USNM).

##### Description.

Eyes in dorsal view not or only slightly bulging beyond temples, temples not receding. Clypeus 1.55–1.65 × wider than high, faintly punctate throughout, nearly smooth; completely concealing labrum when mandible closed, ventral margin of clypeus evenly convex, slightly overlapping dorsal margin of mandible when mandible closed. Antenna with 31–33 flagellomeres. Malar sulcus shallow, weak to indistinct, especially ventrally. Mesosoma 1.3 (male) and 1.2 (female) × longer than high. Pronotum laterally with vertical groove usually weakly crenulate dorsally, distinctly crenulate ventrally, varying from smooth to weakly wrinkled medially, not only faintly and incompletely margined anteriorly by carina. Propodeum unsculptured, with a few weak carinulae along posterior margin, especially medially. Fore wing 3RSa/2RS ratio highly variable, 1.6 (male) and 1.75–1.9 (female) × longer than sinuate 2RS; (RS+M)a varying from weakly sinuate to nearly straight. T1 2.3–2.4 (female) × wider at apex than at base, 1.1 (male) and 0.85–1.0 (female) × as long as apical width; smooth, unsculptured basally, densely and distinctly striate to strigose over apical 0.75 in female, sculpture weaker and less extensive in male; dorsal carina distinct basally, extending to apex but weaker and largely obscured by sculpture posteriorly. Ovipositor (total length) approximately 3.2–3.4 × longer than mesosoma; ovipositor sheath approximately 2.5–2.6 × longer than mesosoma. Head color as in *Opius danielsae*. Mesosoma nearly identical in color to *Opius danielsae* except with two orange spots on either side of metascutellum, an orange spot immediately dorsad midcoxa, and the subalar ridge entirely orange. T1 black; remaining terga mostly brown in male; female with most of T2+3 dark brown, T2 with anterior-lateral corner containing spiracle yellow, T3 with narrow yellow band along posterior margin; T4–6 dark brown anteriorly, yellow posteriorly, with median hyaline patch along anterior margins and narrow hyaline margin posteriorly. Fore and mid tibiae and all femora pale yellow; hind tibia anteriorly mostly yellow tending to infumate dorsal-anteriorly, basal 0.2 dark brown, and apical 0.5–0.6 posteriorly brown. Body length 2.75 mm (male), 2.9–3.0 mm (female); wing length 3.4 mm (male), 3.7–3.9 mm (female); mesosoma length 1.05 mm (male), 1.1–1.15 mm (female). Otherwise having all the characteristics described above for the *baderae* species group.

##### Diagnosis.

This species is nearly identical to *Opius danielsae*, but is smaller and the metasomal color pattern differs, with distinctive transverse dark and white bands in *Opius gabriellae*.

##### Biology.

The fly host reared from *Senecio iodanthus* Greenm. is an apparently undescribed species of *Campiglossa* Rondani (a senior synonym of *Paroxyna* Hendel). The rate of parasitism by the opiine was 15%, but many chalcidoids were also reared from this sample of flower heads and at least some of them likely attacked the tephritid. One of the opiines was reared from a segregated puparium of *Campiglossa*, while the remaining two were reared from flower heads. The single non-paratype specimen from *Montanoa frutescens* Mairet ex DC was reared from an undescribed species of *Neotephritis* Hendel along with 17 flies of this tephritid species. *Senecio iodanthus* is a member of the Asteraceae as is *Montanoa frutescens*.

##### Etymology.

This species is named for Gabriella Vasquez, daughter of the senior author.

##### Remarks.

The single female reared from a capitulum of *Montanoa frutescens* appears identical to the material reared from *Senecio iodanthus* except for a slightly smaller brown spot medially on the upper face. We have explicitly excluded the wasp reared from *Montanoa frutescens* from the paratype series because the host fly and plant differ, and thus it was not used to prepare the formal description.

Ovipositor lengths are reasonable approximations since the base of the ovipositor is evident in both females, protruding against the sternites. The second submarginal cell of the male specimen is shorter and taller than in the females, but more specimens are needed to confirm this as evidence of sexual dimorphism. In *Opius gabriellae*, the male is smaller than the females but in *Opius danielsae*, the females are smaller.

#### Opius godfrayi species group

**Description.** Head: Occipital carina broadly absent middorsally, extending laterally from base of mandible to at least mid eye height, often to dorsal margin of eye in lateral view; joining hypostomal carina ventrally distinctly above base of mandible, merged carina continuing to base of mandible as tall flange bordering a shallow depression on gena laterally. Malar space large, at least as long as basal width of mandible. Clypeus tall, ventral margin thin, sharp, weakly convex, truncate or weakly concave in anterior view, labrum partly exposed between clypeus and mandibles when mandibles closed; clypeus weakly to distinctly protruding in profile, without horn or spine-line protrusions. Mandible about as in *baderae* species group but with dorsal margin slightly to distinctly curved, mandible usually more distinctly narrowed apically. Maxillary palp distinctly longer than head height. Antenna longer than body, with at least 40 flagellomeres, first flagellomere distinctly longer than second. Face, gena, and frons as in *baderae* species group.

Mesosoma: Pronotum dorsally a flat, narrow band, enlarged pronope absent; pronotum laterally with narrow, polished, unsculptured band bordering anterior margin separated along its full length from large, triangular, polished, unsculptured posterior portion by distinct groove, groove prominently carinate along its entire anterior margin. Propleuron without oblique carina or groove dorsad propleural flange. Mesoscutum elevated anteriorly relative to pronotum, with distinctly sloping anterior declivity; largely bare, with decumbent white setae densely covering lateral portions of anterior declivity up to base of notaular depression, more sparsely setose medially on declivity and along lateral margin between notaulus and tegula, row of shorter, weakly decumbent setae scattered in decreasing density along traces of notaulus to posterior margin; without midpit posteriorly; notaulus about as in *baderae* species group, not reaching level of tegula but slightly more attenuate posteriorly in most individuals; supramarginal carina absent. Scuto-scutellar sulcus densely crenulate, very narrow, at least 8 × wider than mid length. Scutellar area and mesopleuron as in *baderae* species group. Midventral longitudinal sulcus of mesothorax finely but distinctly crenulate anteriorly, more weakly sculptured to smooth posteriorly. Metapleuron unsculptured medially; median pit adjacent anterior margin and dorsal pit at posterior margin both relatively small. Propodeal spiracle closer to anterior than posterior margin; pleural sulcus distinct from spiracle to posterior margin; propodeum largely unsculptured, without median carina or median areola, usually with pair of short lateral-median longitudinal carinae apically. Metasomal and hind coxal cavities confluent: not separated by sclerotized bridge.

Legs and wings: Hind tibia without basal carina. Wings hyaline. Fore wing stigma narrow, tapered, with r arising basad its midpoint and separated from extreme base of stigma by at least its own length; 1RS short, 1M 5-8× longer than 1RS; 2RS present, weakly to distinctly sinuate, not thickened medially, 3RSa at least 1.45 × longer than 2RS, 3RSb extending to apex of wing or nearly so, distinctly bowed, not foreshortened; 2nd submarginal cell narrowing distally, height at r 1.5–1.75 × height at r-m; m-cu postfurcal; 2CUa distinctly shorter than 2cu-a, 2CUb thus arising anteriorad middle of distal margin of 1st subdiscal cell; 1st subdiscal cell weakly expanded distally; shortest distance between anal vein and ventral wing margin equal to 1–2 × width of anal vein. Hind wing with 3 hamuli; RS largely spectral, sometimes weakly pigmented basally, much weaker than M; M distinct, usually tubular and well-pigmented over at least basal 0.3, sometimes nebulous basally; m-cu completely absent.

Metasoma: S1 and T1 as in *baderae* species group. T2 and remaining terga unsculptured. Hypopygium collapsed on only known female specimen; details of hypopygium and ovipositor not visible.

**Diagnosis.** Unlike most other Opiinae, the occipital and hypostomal carinae meet well above the base of the mandible in members of the *godfrayi* species group, continuing to the mandible as a single, flange-like ridge. They would thus key to *Apodesmia* Foerster in Li et al. (2013), though differing notably from the type species of *Apodesmia* in the absence of a midpit on the mesoscutum and absence of a sculptured precoxal sulcus, among other features. Some members of the *godfrayi* species group will key to *Opius* (*Pendopius* Fischer) in the subgeneric keys of Fischer (1972, 1999), but differ from the type species of *Pendopius* in lacking a basal tooth or lobe on the mandible. Others will key to *Opiothorax* Fischer, the type species of which similarly has a basal lobe on the mandible.

**Remarks.** While the fusion of the occipital and hypostomal carina ventrally suggests a relationship to *Apodesmia*, sculptural characteristics of the mesosoma (especially notauli, pronotum, precoxal sulcus, and propodeum) and metasoma (T1) as well as the position of fore wing 2CUb suggest a closer relationship to members of the *baderae* species group. The hypothesis of a more distant relationship to *Apodesmia* is also supported by differences in the way in which the hypostomal and occipital carinae converge in *Apodesmia* vs members of the *godfrayi* species group. Three species are described here as members of this species group: *Opius godfrayi*, *Opius marshi*, and *Opius nablus*.

There is a gradation among species of the *godfrayi* group in the amount of curvature and deflection of the dorsal margin of the mandible, affecting the degree of exposure of the labrum. The labrum is very narrowly exposed in *Opius godfrayi*, for example, but broadly exposed in *Opius marshi*. Comparisons must be done with individuals in which the mandibles are completely closed since partial closure will also affect the amount of exposure of the labrum.

Three additional members of this species group are in the TAMU collection, represented by unreared singletons from Mexico, Costa Rica, and Panama, respectively. They vary from one another and from the species described here in color pattern, degree of exposure of the labrum, and development of the notaulus.

#### Opius godfrayi

Wharton sp. n.

http://zoobank.org/92BD7106-9CC5-418A-9208-7968D5ED5EFD

http://species-id.net/wiki/Opius_godfrayi

Figs 3 , 23 , 55–58

##### Type locality.

Mexico, Morelos, Lago de Zempoala.

##### Type material.

Holotype. Male (UNAM), first label, first line: MEXICO: Morelos second line: Lago de Zempoala third line: 23–25.ix.1991 fourth line: A. L. Norrbom, # 50 Second label, first line: reared ex. stem gall second line: *Dahlia imperialis* third line: Roezl. (91M16A) Third label, first line: reared ex. puparium second line: *Eutreta christophe* third line: (Tephritidae).

**Paratypes:** 6 males, same data as holotype (TAMU, USNM).

##### Description.

*Male*. Eyes in dorsal view slightly bulging beyond temples, temples not or only very weakly receding. Clypeus 1.6–1.75 × wider than high, weakly punctate throughout; hemispherical or nearly so with epistomal sulcus even rounded; nearly flat in profile, very weakly protruding ventrally; ventral margin very weakly convex, nearly truncate in anterior view with mandibles weakly deflected, exposing very small portion of labrum. Antenna with 41–43 flagellomeres. Malar sulcus distinctly impressed throughout, deeper near eye. Mesosoma 1.3–1.4 × longer than high. Pronotum laterally crenulate along most or all of posterior side of distinctly elevated vertical carina, sculpture weaker, occasionally evanescent medially; carina extending full length of sclerite in lateral view. Notaulus comma-shaped: a short, curved groove extending posteriorly from a rounded pit, deep anteriorly, increasingly shallow posteriorly, not margined anteriorly by carinae. Setae scattered along traces of notaulus very short and widely spaced, mostly absent over posterior 0.5 of mesoscutum. Metapleuron with median pit adjacent anterior margin not directly connected to dorsal pit at posterior margin by a sulcus; ventral margin without well-developed spine anteriorly, at most with ventral carina weakly, unobtrusively expanded anteriorly. Propodeum with rugulose area mesal-ventrally of spiracle and weakly punctate to rugulose anteriorad ends of short but distinct lateral-median carinae, otherwise mostly smooth and polished. 3RSa 1.45–1.65 × longer than sinuate to strongly sinuate 2RS; (RS+M)a usually weakly sinuate, rarely strongly so. T1 1.9–2.1 × wider at apex than at base, 0.9–1.05 × as long as apical width; smooth, unsculptured basally, striate to strigose over weakly elevated apical 0.6–0.7, more finely and irregularly sculptured apical-laterally; dorsal carina distinct basally, extending to apex but largely obscured by sculpture posteriorly, indicated only as lateral margin of weakly elevated median area. Color: Head mostly yellow, including face, broad orbital band extending from level of antenna through upper gena; lower orbit, lower gena, malar space, clypeus, and mouthparts (except apical teeth of mandible) whitish; frons medially, continuing as a broad band through ocellar field, adjacent portion of vertex, and dorsal half of occiput dark brown to black. Mesosoma black to dark red-brown except yellow as follows: propleuron, pronotum dorsally, anterior polished band of pronotum laterally, much of mesoscutum, at least lateral margin of scutellar triangle and posterior polished band, a pair of spots on either side of metanotal midline and entire posterior margin of metanotum, subalar elevation, somewhat rectangular spot on mesopleuron immediately dorsad mid coxa, ventral midline of mesothorax, and at least ventral part of metapleuron; scutellum medially, at least part of axilla, scuto-scutellar sulcus, and metapleuron dorsally usually light brown, rarely entirely yellow; mesoscutum variable: from mostly yellow with narrow dark brown to black streak along posterior-lateral margin extending from tegula to axilla and faintly infumate medially (Fig. 58) to much darker with three large dark markings anterior-medially and posterior-laterally (about as in Fig. 45); tegula and basal wing sclerite white. T1 black; T2 with median 0.6–0.7 black, lateral margin including spiracle pale yellow; T3-T6 transversely banded black anteriorly, brown medially, white to hyaline posteriorly, median white band usually visible along anterior margin; T7 mostly white, usually weakly spotted with brown medially. Fore and mid tibiae and femora pale yellow; hind femur with pale brown subapical spot on anterior and posterior face, otherwise pale yellow; hind tibia brown with basal 0.2 dark brown. Body length 3.6–4.2 mm; wing length 4.25–4.65 mm; mesosoma length 1.35–1.5 mm. Otherwise having all the characteristics described above for the *godfrayi* species group.

**Diagnosis.** Of those opiines in which the occipital and hypostomal carinae are united before reaching the mandible, this species is most readily characterized by the relatively concealed labrum, with only a small portion exposed between the ventral margin of the clypeus and the dorsal margin of the mandibles. *Opius godfrayi* also has a darker mesopleuron than both *Opius marshi* and *Opius nablus*, the only other members of this species group described here. *Opius godfrayi* could key to *Opius (Pendopius) vinoanus* Fischer in Fischer (1977, 1983), but the latter has a darker mesoscutum and a sculptured propodeum.

##### Biology.

All members of the type series were reared from puparia of *Eutreta christophe* that were reared from stem galls of *Dahlia imperialis*. Three flies emerged from this sample, resulting in 70% parasitism by *Opius godfrayi*. Three other opiine species were reared from flower heads of this plant at the same locality. Details are given under the biology section of *Opius danielsae* above. This is a new host plant record for the fly.

##### Etymology.

This species is named for Charles Godfray for his many contributions to parasitoid ecology and especially for improving our understanding of host relationships in leaf miner parasitoids.

##### Remarks.

This species is known only from males. The color pattern on the mesoscutum is remarkably variable. On the head, the broad band on the frons extending through the ocellar field and half way down the occiput is only slightly variable, with the band narrowed on the vertex in one specimen and extending variously to or between the antennae. The propodeum shows more evidence of sculpture in this species than other species of either the *baderae* or the *godfrayi* groups, but is unusually variable in extent. The propodeum is largely smooth and polished even in the most heavily sculptured specimen, where rugulose lines separate the large, median polished area from a narrower lateral polished area.

#### Opius marshi

Wharton sp. n.

http://zoobank.org/FBB8209B-1284-41FE-8105-DDE882991A2A

http://species-id.net/wiki/Opius_marshi

Figs 24 , 59–62

##### Type locality.

Mexico, Chiapas, south slope Volcan Tacaná, Chiquihuites.

##### Type material.

Holotype. Female (UNAM), first label, first line: MEXICO: Chiapas second line: Chiquihuites, ~15°05'N third line: 92°06'W, NW Union Juarez Second label, first line: S slope Volcan Tacana, second line: 1800–2000 m, 31.X.1993 third line: A.L.Norrbom & C.Estrada Third label, first line: reared ex. stem gall second line: *Squamopappus skutchii* third line: (Blake) Janson, Harriman fourth line: & Urbatsch (93M11) Fourth label, first line: reared ex stem second line: gall of *Eutreta apicata* third line: (Tephritidae).

**Paratypes:** 1 male, same data as holotype (TAMU). 1 male, Mexico: Chiapas, between Union Juarez & Chiquihuites, ~15°05'N, 92°05'W, S slope Volcan Tacana, 1500–1800 m, 4.xi.1993, A. L. Norrbom & C. Estrada, 93M22, reared from gall of *Eutreta apicata* Hering on stem of *Podachaenium eminens* (Lag.) Sch.Bip. (USNM).

##### Description.

Eyes in dorsal view bulging beyond temples, temples weakly but distinctly receding. Clypeus 1.75–1.85 (male) and 2.0 (female) × wider than high, weakly punctate throughout, more deeply punctate along ventral margin; very weakly triangular, nearly hemispherical with epistomal sulcus almost evenly rounded; nearly flat in profile dorsally, ventral margin weakly but distinctly protruding, truncate in anterior view with mandibles deflected, exposing most of labrum. Antenna with 43–45 (male) and 46 (female) flagellomeres. Malar sulcus impressed throughout, deeper near eye, weak to nearly absent near mandible. Mesosoma 1.35–1.4 × longer than high. Pronotum laterally weakly crenulate dorsally and ventrally along posterior side of distinctly elevated vertical carina, broadly absent medially, sculpture more distinct in largest specimen. Notaulus about as in *Opius godfrayi*, but curved groove not ending in distinct pit at anterior end. Setae scattered along traces of notaulus longer and denser over anterior 0.7 of mesoscutum. Metapleuron with median pit adjacent anterior margin not directly connected to dorsal pit at posterior margin by a sulcus; ventral margin without well-developed spine anteriorly, but with ventral carina angled at 90 degrees anteriorly and weakly expanded as a flange. Propodeum with small rugulose area mesal-ventrad spiracle, a few irregular, deep punctures anteriorad ends of short lateral-median carinae, and a few weak carinulae along posterior margin, otherwise mostly smooth and polished; sculpture more distinct in largest specimen. 3RSa 1.55–1.75 × longer than sinuate to strongly sinuate 2RS; (RS+M)a weakly sinuate. T1 1.75–2.0 (male) and 2.15 (female) × wider at apex than at base, 0.95–1.0 × as long as apical width; T1 smooth, unsculptured basally and apical-laterally, striate to finely strigose over middle portion of apical 0.4–0.5, more extensively sculptured in female: striate to strigose apical-laterally and over apical 0.5; dorsal carina sharply elevated basally, forming almost a tuberculate angle as it extends posteriorly, extending to apex but broader, rounded, gradually becoming indistinct over apical 0.5. Color: Head adjacent eyes yellow above, fading to whitish below, especially on face laterad clypeus and mouthparts (except apical teeth of mandible dark); frons medially, continuing posteriorly as a band through ocellar field and onto vertex dark brown to black, with similarly dark transverse band across middle of occiput, enlarged at each end; face with infumate spot dorsal-medially or broader dark band extending to epistomal sulcus; head darker in female than male. Mesosoma of male yellow-orange with propodeum, midline of metanotum, and small spot along lateral margin of mesoscutum immediately anteriorad axilla black; small to large spot at apex of scutellum and large spot dorsal-medially on pronotum laterally dark red-brown; parascutellar field, posterior-lateral field of metanotum, and mesopleuron ventrally anteriorad mid coxa infumate (as a spot in one specimen and a longer streak in the other); tegula and basal wing sclerite white, propleuron and polished, anterior margin of pronotum laterally white or nearly so; female darker, with mesopleuron mottled yellow and brown and scutellar and metanotal areas completely suffused with brown. Metasomal terga black with hyaline posterior margin of T3 and following usually visible, T3–T6 medially with white band along anterior margin, visible portion of T7 white in female. Fore and mid tibiae and femora whitish to pale yellow; hind femur with pale brown subapical spot only on anterior face, otherwise pale yellowish white; hind tibia brown with basal 0.2 dark brown, usually pale brown medially, especially on posterior face. Body length 4.0–4.75 mm; wing length 4.75–5.4 mm; mesosoma length 1.65–1.9 mm. Otherwise having all the characteristics described above for the *godfrayi* species group.

##### Diagnosis.

*Opius marshi* belongs to the *godfrayi* species group based on the fusion of the hypostomal and occipital carinae ventrally. It differs from *Opius godfrayi* by the more broadly exposed labrum and from *Opius nablus* by the more deeply incised notauli.

##### Biology.

*Opius marshi* was reared from tephritid stem galls made by *Eutreta apicata* on two different plants in the family Asteraceae. Sample 93M11 from *Squamopappus skutchii* produced two wasps and one fly (66.7% parasitism), and sample 93M22 from *Podachaenium eminens* yielded one fly and one wasp (50% parasitism). Both are new host plant records for *Eutreta apicata*.

##### Etymology.

This species is named for Paul Marsh for his contributions to braconid taxonomy and his assistance in facilitating the work on this material.

##### Remarks.

The male wasp from *Podachaenium eminens* is larger than the male from *Squamopappus skutchii* and the dark markings on the head are not quite as extensive though of the same pattern. The base of the ovipositor and ovipositor sheath are too well-concealed in the only female specimen to provide a useful approximation of total length for this species.

#### Opius nablus

Wharton sp. n.

http://zoobank.org/7214B3DC-BC43-4DBE-A88B-A3B1BE52FFBB

http://species-id.net/wiki/Opius_nablus

Figs 25 , 63–66

##### Type locality.

Guatemala, Sacatepequez, 3–6 km west of San Miguel Dueñas.

##### Type material.

Holotype. Male (USNM), first label, first line: GUATEMALA: Sacatepequez second line: San Miguel Duenas, 3–6 km third line: W, 17.X.1990, A. L. Norrbom Second label, first line: reared ex. stem gall second line: of Tephritidae sp. on third line: *Verbesina fraseri* (90G8) Third label: ALN–3.

##### Description.

*Male*. Eyes in dorsal view bulging beyond temples, temples weakly but distinctly receding. Clypeus 2.1 × wider than high, weakly punctate throughout; weakly triangular in outline, epistomal sulcus not even rounded; nearly flat in profile, very weakly protruding ventrally; ventral margin very weakly concave in anterior view with mandibles deflected, exposing substantial portion of labrum. Antenna with 41 flagellomeres. Malar sulcus impressed throughout, deeper near eye. Mesosoma 1.4 × longer than high. Pronotum laterally completely unsculptured or nearly so along posterior side of distinctly elevated vertical carina. Notaulus a short, curved, shallow groove not reaching anterior margin, not margined anteriorly by carinae; associated setae as in *Opius marshi*. Metapleuron with median pit adjacent anterior margin connected to dorsal pit at posterior margin by a very weak sulcus; ventral margin without well-developed spine anteriorly, at most with ventral carina weakly, unobtrusively expanded anteriorly. Propodeum medially smooth, polished, with a pair of short lateral-median carinae; weakly rugulose along lateral margin, especially in vicinity of spiracle. Fore wing 3RSa 1.55 × longer than strongly sinuate 2RS; (RS+M)a very weakly sinuate. T1 2.1 × wider at apex than at base, 1.1 × longer than apical width; smooth, unsculptured basally and apical-laterally, striate to strigose over middle portion of apical 0.5; dorsal carina distinct basally, extending towards but not obviously attaining apex, weak and obscured by sculpture posteriorly. Color: Head with yellow orbital band extending posteriorly from torulus to gena at mid eye height, gena ventrally, lower occiput, malar space, orbital band between torulus and malar sulcus, clypeus, and mouthparts (except dark apical teeth of mandible) white; broad band extending from epistomal sulcus through dorsal half of occiput dark brown to black, the dark color extending slightly onto upper gena. Mesosoma similar in color to *Opius marshi*: pale yellow-orange except nearly all of pronotum dorsally and laterally, irregular streak ventral-laterally on mesopleuron extending between fore and mid coxae, scuto-scutellar sulcus, median longitudinal band on entire scutellum, and most of remaining parts of scutellar and metanotal area (except for a pair of yellow spots on either side of midline) brown. Metasomal tergal color and leg color as in *Opius marshi*. Body length 4.0 mm; wing length 4.35 mm; mesosoma length 1.45 mm. Otherwise having all the characteristics described above for the *godfrayi* species group.

**Figures 25–28.**
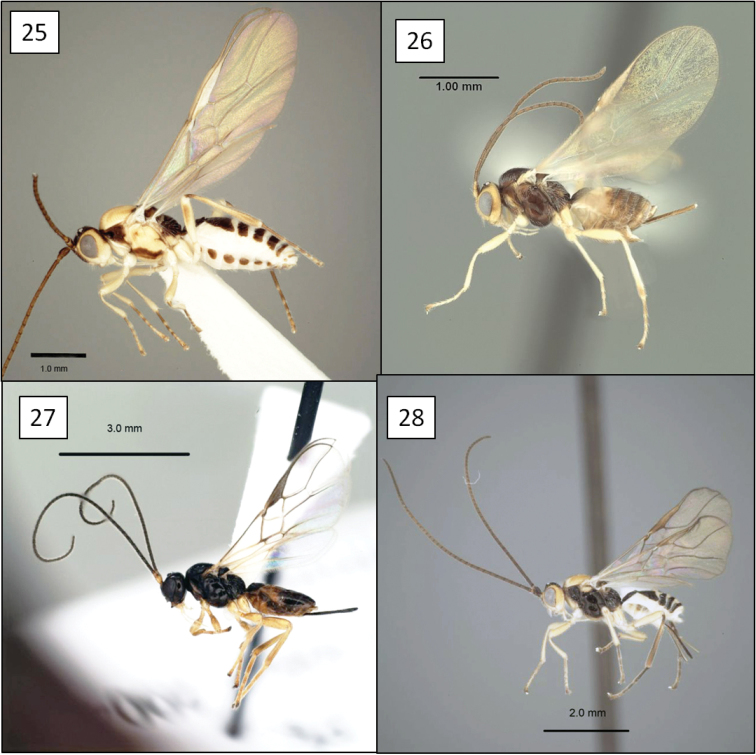
*Opius* spp., habitus. **25** *Opius nablus* Wharton, sp. n. **26** *Opius nympha* Fischer **27** *Opius peleus* Fischer **28** *Opius pipitae* Wharton, sp. n.

##### Diagnosis.

This species is nearly identical to *Opius marshi* with slightly darker head and lighter hind tibia than males of that species. Perhaps more importantly, the notaulus is shorter and less distinctly impressed in *Opius nablus* relative to *Opius marshi* and *Opius godfrayi*.

##### Biology.

The only known specimen was reared from a stem gall on the asteracean plant *Verbesina fraseri* Hemsl. No flies were reared from this sample, so the tephritid host is unknown.

##### Etymology.

The species name is an arbitrary combination of letters.

##### Remarks.

This species is known from a single male specimen. In this specimen, hind wing M is more weakly developed than in other members of this species group, but the difference is not great.

#### Opius nympha

Fischer

http://species-id.net/wiki/Opius_nympha

Figs 4 , 26 , 67–70

Opius nympha Fischer, 1968: 34–35, 40–41 (key), 111–113 (description). Holotype female in CNC (examined).
Opius nympha : Fischer 1971: 92 (catalog).
Opius (Thoracosema) nympha : Fischer 1977: 409–410, 436–437 (key, redescription); Yu et al. 2005, 2012 (electronic catalogs).

##### Type locality.

Mexico, Mexico, Toluca.

##### Type material.

Holotype. Female (CNC), first label, first line: Toluca, 10 Mi. E., second line: 8900’, Mexico, Mex., third line: 31–VII–1958, fourth line: J. G. Chillcott.

##### Other specimens examined.

Mexico: 5 females, 10 males, Distrito Federal, Rt. 95 between Km 42 and 43, 1 km N La Cima, 8.viii.1989, A.L. Norrbom, reared ex. *Paroxyna* from flowers of *Senecio sanguisorbae* DC. (89M3) (TAMU, USNM); 2 females, 4 males, same host and host plant data but Morelos, Parque Lago de Zempoala, 9–11.viii.1989, A.L. Norrbom (89M9) (TAMU, USNM); 5 females, 5 males, same locality and collector but 23–25.ix.1991, reared ex. *Paroxyna* sp. from capitulae of *Roldana lineolata* (DC.) H. Rob. & Brettell (91M11) (TAMU, USNM); 2 females, Mexico, Rt. 890 Km 9 area, 6 km W Lago Zempoala, 2.x.1991, A.L. Norrbom, reared ex. *Paroxyna* sp. from capitulae of *Senecio iodanthus* Greenm. (91M33) (TAMU, USNM); 2 females, Mexico, Parque Popo Izta, Rt. 451 (Amecameca Cholula), Km 17.6, 13.viii.1989, A.L. Norrbom; reared ex. *Paroxyna* from stems of *Barkleyanthussalicifolius* (Kunth) H. Rob. & Brettell (89M1) (TAMU, USNM).

##### Diagnosis.

Temple narrow, eye about 2.0–2.5 × longer than temple in both dorsal and lateral view. Clypeus somewhat crescentic, ventral margin weakly protruding in lateral view, sharp, truncate medially, curving ventrally near lateral margins; labrum broadly exposed. Malar space about as long as basal width of mandible, malar sulcus complete, deeply incised throughout. Occipital carina widely absent dorsally, the gap greater than distance between eyes in dorsal view, carina present, well developed laterally, widely separated from hypostomal carina at base of mandible. Antenna with 20–21 (female) and 22–24 (male) flagellomeres. Pronotum dorsally narrow, with median pit; laterally without vertical carina adjacent median vertical groove. Mesoscutum with deep, vertical anterior declivity; notaulus a short, deep impression barely extending posteriorly beyond anterior declivity, continuing to posterior margin of mesoscutum only as a narrow band of setae, disc of mesoscutum otherwise bare, without midpit posteriorly; supramarginal carina well-developed. Precoxal sulcus short, not extending to anterior or posterior margins of mesopleuron, nearly always (95%) crenulate. Propodeum largely smooth, polished, with some rugulose sculpture medially adjacent posterior margin; setose laterally. Hind tibia without basal carina. Fore wing stigma wedge-shaped, gradually merging with R1 distally, r arising from basal 0.25; 3RSa 1.6–1.95 (female) and 1.5–1.85 (male) × longer than 2RS; m-cu distinctly postfurcal; 2CUb arising distinctly below middle of distal margin of 1st subdiscal cell. Hind wing RS absent; m-cu present, extending 0.3–0.5 distance to wing margin as a crease, usually weakly pigmented basally. T1 with laterope but without dorsope; dorsal carinae distinct to level of spiracle, nearly absent posteriorly, not reaching posterior margin, rugulose to nearly smooth between carinae. T2 and following without sculpture. Ovipositor (total length) 1.4 × longer than mesosoma; ovipositor sheath 0.9 × length of mesosoma. Color: head yellow, usually with faint infumate spots around dark ocellar triangle and posteriorad middle of eye; meso- and metasoma dark brown with T2+3, small spot surrounding propodeal spiracle, and some to all of pronotum laterally yellow; male often with yellow markings on mesopleuron, sometimes extensively; wings hyaline.

##### Biology.

All of the specimens were reared from species of the tephritid genus *Campiglossa* (currently treated as a senior synonym of *Paroxyna*, the genus name indicated on the labels as noted above under specimens examined). The vast majority of the specimens were reared from flower heads, but two of the specimens were from stems containing flies of this genus. Wasps and flies were reared from four species of Asteraceae, representing three or four closely related genera [*Senecio sanguisorbae* (DC.) C. Jeffrey is also known as *Packera sanguisorbae* (DC.) C. Jeffrey]. Plant host names are given in the materials examined section. One of the samples yielded 28 flies and 5 braconids (15% parasitism by *Opius nympha*).

##### Remarks.

Fischer (1968, 1977) provides a detailed description and keys for this species, all in German. The above diagnosis is intended primarily to highlight features useful for separating *Opius nympha* from the other species of *Opius* s.l. treated here. This species is readily distinguished from members of the *baderae*, *godfrayi*, and *pipitae* species groups by the distinctly lower position of 2CUb arising from the distal side of the 1st subdiscal cell. From the remaining species of *Opius* described or otherwise treated in this publication, O. *nympha* can be differentiated by the absence of a mesoscutal midpit, the presence of a weakly sculptured precoxal sulcus, and the lack of rugose or carinate sculpture medially on the propodeum.

Fischer (1977) placed *Opius nympha* in the subgenus *Thoracosema* Fischer, characterized by reduced propodeal sculpture. The precoxal sulcus is very weakly sculptured in a few of the specimens, and these would likely run instead to *Phaedrotoma* in Fischer (1977). *Opius nympha* also superficially resembles species in the Old World genus *Psyttalia* Walker, but differs in such characteristics as the lack of propodeal carinae medially and the presence of a hind wing m-cu in addition to lacking a short T2.

All of the specimens listed in the material examined section appear to represent a single species, with color and sculptural variation as great within each series as it is among the different series. However, there are an additional six specimens from Costa Rica and one from Guatemala (all USNM, all reared from *Campiglossa*) that appear to represent one or more species. They differ slightly from *Opius nympha* in having an extensively rugose propodeum and very slightly longer ovipositor, but because of the sculptured propodeum they would not be placed in the same subgenus as *Opius nympha* in the classification of Fischer (1972, 1977). The morphological variation in the material at hand thus suggests that *Opius nympha* is likely to be just one of several closely related species specializing on *Campiglossa*. Determining whether other members of this group are described or undescribed will require extensive comparisons across several large subgenera in Fischer’s (1972, 1977) classification, the only one providing extensive keys for Neotropical species.

#### Opius peleus

Fischer

http://species-id.net/wiki/Opius_peleus

Figs 2 , 9 , 27 , 71–74

Opius peleus Fischer, 1970: 802–804. Holotype male in AEIC (examined).
Opius peleus : Marsh 1974: 287 (synonymy); Marsh 1979: 210 (catalog).
Opius (Merotrachys) peleus : Fischer 1977: 655, 695–697 (key, redescription); Fischer 1979: 264 (key); Yu et al. 2005, 2012 (electronic catalogs).
Opius telephosi Fischer, 1970: 812–815. Synonymized by Marsh (1974: 287).

##### Type locality.

USA, South Carolina, Pickens County, Wattacoo.

##### Type material.

Holotype. Male (AEIC), data label, first line: Wattacoo, Pickens Co., S. C. second line: V. 27. 61 third line: G. F. Townes.

##### Other specimens examined.

5 females, 2 males, USA: Tennessee, Blount Co., Great Smoky Mountains National Park, Abrams Creek Campground, 3.xi.2003, G.J. Steck & B.D. Sutton, reared ex. *Strauzia intermedia* from root mines of *Rudbeckia laciniata* (FSCA, TAMU); 1 female, same data except 7.viii.2002; 1 female, same data except 5.iii.2004 (FSCA).

##### Diagnosis.

Temple relatively broad, eye about 1.75–2.1 (female) and 1.35–1.6 (male) × longer than temple in lateral view, 1.1–1.3 × longer than temple in dorsal view. Clypeus hemispherical, ventral margin weakly protruding in lateral view, sharp, truncate; mandibles deflected, labrum broadly exposed. Malar space almost as long as basal width of mandible, malar sulcus complete, deeply incised throughout. Mandible broadening basal-ventrally, but without distinctly delineated basal lobe or tooth. Face variable in sculpture, minimally with strigose band along inner margin of eyes. Occipital carina absent dorsally, the gap less than distance between eyes in dorsal view, carina present and well developed laterally, widely separated from hypostomal carina at base of mandible. Antenna with 43–47 (45 in holotype) flagellomeres, apical flagellomere long and conical. Pronotum dorsally narrow, with median pit; laterally with vertical carina adjacent median vertical groove usually present on ventral 0.3; crenulate along posterior margin, medially varying adjacent the margin from largely smooth to extensively rugulose. Mesoscutum with deep, strongly sloping anterior declivity; notaulus a short, very deep impression barely extending posteriorly beyond anterior declivity; disc of mesoscutum largely bare, without midpit posteriorly; supramarginal carina absent, but base of notaulus rugulose. Precoxal sulcus usually distinct as a broad, shallow, impression, short, not extending to anterior or posterior margins of mesopleuron, always unsculptured; mesopleural fovea crenulate along entire posterior margin of mesopleuron. Propodeum rugulose throughout, short median carina sometimes distinct basally. Hind tibia without basal carina. Fore wing stigma broad, wedge-shaped, relatively discrete distally, r arising from middle; 3RSa 1.3–1.45 × longer than 2RS; m-cu usually interstitial, varying from very weakly antefurcal to weakly postfurcal; 2CUb arising distinctly anteriorad middle of distal margin of 1st subdiscal cell. Hind wing RS largely spectral, weakly pigmented basally; m-cu present as a spectral vein extending nearly to wing margin. T1 with broad, deep laterope but without dorsope; dorsal carinae strongly elevated basally, converging to form a deep basal depression, absent posteriorly beyond spiracle; T1 rugose over posterior 0.5. T2 usually with trace of weakly rugulose sculpture, sculpture sometimes not apparent. Ovipositor (total length) 1.4–1.5 × longer than mesosoma; ovipositor sheath 0.9 × length of mesosoma. Color: dark brown to black; mandible reddish yellow to yellow with apical teeth black; scape, pedicel, remaining mouthparts, legs, and usually T2, 3, 7, 8 yellow; wings hyaline.

##### Biology.

All of the specimens were reared from the tephritid *Strauzia intermedia* (Loew) collected from root mines of *Rudbeckia laciniata* L.

##### Remarks.

Fischer (1970, 1977) provides a detailed description and keys for this species, all in German. The above diagnosis is intended primarily to highlight features useful for separating *Opius peleus* from the other species of *Opius* s.l. treated here. This species is readily distinguished from *Opius nympha*, *Opius taramegillae*, and members of the *baderae*, *godfrayi*, and *pipitae* species groups by the presence of extensive sculpturing on the propodeum. *Opius yoderi*, the only other species described here with an extensively sculptured propodeum, has a densely furry mesoscutum (Fig. 89).

Fischer (1977) placed *Opius peleus* in the genus *Merotrachys*, which he defined in part by the presence of sculpture on the second metasomal tergum. In the holotype, the striate sculpture on T2 is more distinct than in the specimens reared from *Strauzia* in Tennessee. Sculpture is variable in the Tennessee specimens, with some individuals exhibiting virtually no obvious sculpture while others are weakly striate or punctato-striate. In either case, the sculpture in *Opius peleus* is distinctly different from that found in members of the *ingenticornis* species group, most of which have previously been included in *Merotrachys* (Wharton et al. 2013). *Opius peleus* also lacks the large pronope and complete dorsal carinae on T1 characteristic of members of the *ingenticornis* group.

*Opius peleus* is very similar to *Opius antrimensis* Fischer but the latter is only 0.5–0.7 × the size of *Opius peleus*. Details of the facial sculpture, which Fischer (1977, 1979) emphasized as diagnostic for *Opius peleus* in his keys, are difficult to discern in the holotype and only known specimen of *Opius antrimensis*. The similarity between these two species suggests the possibility that *Opius antrimensis* also attacks tephritids or other maggots feeding in roots or lower portions of stems.

#### Opius pipitae species group

**Description.** Head: Occipital carina broadly absent middorsally, extending laterally from base of mandible to at least mid eye height, often to dorsal margin of eye in lateral view; widely separated from hypostomal carina ventrally; hypostomal carina elevated as flange near base of mandible, flange clearly visible in lateral view protruding beyond the slightly reflected occipital carina. Malar space large, approximately as long as basal width of mandible. Clypeus tall, ventral margin thin and sharp, weakly convex, truncate, or weakly concave in anterior view, labrum partly exposed between clypeus and mandibles when mandibles closed; clypeus distinctly protruding in profile, without horn or spine-line protrusions. Mandible narrowed apically, more distinctly so than in *baderae* species group, apical teeth slightly twisted with ventral tooth smaller and more posteriorly positioned. Maxillary palp at least as long as height of head.

Mesosoma: Pronotum dorsally a flat, narrow band, enlarged pronope absent, though a shallow median dimple may be present; pronotum laterally with narrow, polished band bordering anterior margin separated along its full length from large, triangular, polished, unsculptured posterior portion by distinct groove, groove sculptured, at least in part, and at least partially carinate along anterior margin. Propleuron without oblique carina or groove dorsad propleural flange. Mesoscutum elevated anteriorly relative to pronotum, with distinctly sloping anterior declivity; largely bare, with decumbent white setae densely covering lateral portions of anterior declivity up to base of notaular pit, more sparsely setose medially on declivity and along lateral margin between notaulus and tegula, row of shorter, weakly decumbent setae scattered in decreasing density along traces of notaulus to posterior margin; without midpit posteriorly; notaulus as in *godfrayi* species group; supramarginal carina absent. Scuto-scutellar sulcus densely crenulate, narrow, 6–7 × wider than mid length. Scutellar area and mesopleuron as in *baderae* and *godfrayi* species groups. Midventral longitudinal sulcus of mesothorax finely but distinctly crenulate. Metapleuron unsculptured medially; median pit adjacent anterior margin and dorsal pit at posterior margin both relatively small, not directly connected medially by a sulcus; ventral margin without well-developed spine anteriorly, at most with ventral carina weakly, unobtrusively expanded anteriorly. Propodeal spiracle closer to anterior than posterior margin; pleural sulcus distinct from spiracle to posterior margin; propodeum largely unsculptured, without median carina or median areola, usually with pair of short lateral-median longitudinal carinae apically. Metasomal and hind coxal cavities confluent: not separated by sclerotized bridge.

Legs and wings: Hind tibia without basal carina. Wings hyaline. Fore wing stigma narrow, tapered, with r arising basad its midpoint and separated from extreme base of stigma by at least its own length; 1RS short, 1M 6–9x longer than 1RS; 2RS present, weakly to distinctly sinuate, not thickened medially, 3RSa 1.35–1.55x longer than 2RS, 3RSb extending to apex of wing or nearly so, not foreshortened; 2nd submarginal cell narrowing distally; m-cu postfurcal; 2CUa distinctly shorter than 2cu-a, 2CUb thus arising anteriorad middle of distal margin of 1st subdiscal cell; 1st subdiscal cell weakly expanded distally; shortest distance between anal vein and ventral wing margin equal to 1–2x width of anal vein. Hind wing with 3 hamuli; RS largely spectral, sometimes weakly pigmented basally, much weaker than M; M distinct, usually nebulous and pigmented over at least basal 0.5; m-cu completely absent.

Metasoma: S1 and T1 as in *baderae* species group. T2 and remaining terga unsculptured. Hypopygium broadly triangular, pointed apically. Ovipositor with very weak dorsal node near apex.

**Diagnosis.** The species in this group are similar to members of the *baderae* and *godfrayi* species groups in general appearance, reduced propodeal sculpture, absence of a midpit on the mesoscutum, absence of sculpture within the precoxal sulcus, and the notably anterior position of 2CUb along the distal margin of the 1st subdiscal cell. As in members of the *godfrayi* species group, the labrum is exposed in the gap between the ventral margin of the clypeus and the dorsal margin of the mandibles when the latter are closed. Unlike members of the *godfrayi* species group, however, the occipital and hypostomal carinae are widely separated ventrally, a characteristic of members of the *baderae* species group.

**Remarks.** Three species are included in the *Opius pipitae* species group: *Opius pipitae*, *Opius stecki*, and *Opius townesi*. These species will key to either *Pendopius* or *Opiothorax* in the subgeneric classification of Fischer (1972, 1977) and to *Adontopius* Fischer in Fischer (1999). In Li et al. (2013), they key to *Phaedrotoma*. In members of the *pipitae* species group, the vertical carina on the pronotum laterally is very well developed and the pleural sulcus is distinct both anterior and posterior to the propodeal spiracle.

#### Opius pipitae

Wharton sp. n.

http://zoobank.org/5340A9BE-961E-4673-8C93-2C69A3B71E00

http://species-id.net/wiki/Opius_pipitae

Figs 28 , 75–78

##### Type locality.

Mexico, Morelos, Huitzilac.

##### Type material.

Holotype. Female (UNAM), first label, first line: MEXICO: Morelos second line: Huitzilac, 22.X. third line: 1991, A.L.Norrbom Second label, first line: reared ex. lateral second line: stem gall on *Montanoa* third line: *frutescens* (91M5A) Third label, first line: ex. Tephritidae second line: n. gen., n. sp.

**Paratypes:** 1 female, same data as holotype (USNM). 2 females, 3 males, Mexico: Morelos, 2 km W of Huitzilac, 29.ix.1987, A.L. Norrbom & V. Hernandez O., reared ex. stem gall *Montanoa frutescens* Mairet ex. DC. (M–21) (TAMU, USNM). 2 females, Mexico: Morelos: ridge above Sto. Domingo Ocotitlan, 21.ix.1991, A.L. Norrbom & L. Quiroz, reared ex. lateral stem gall on *Montanoafrutescens* (91M5) (TAMU, USNM).

##### Description.

Eyes in dorsal view very slightly bulging beyond temples, temples weakly but distinctly receding. Clypeus 1.7–1.9 × wider than high, weakly punctate, more deeply and densely so along ventral margin; nearly hemispherical in outline with epistomal sulcus almost evenly rounded, slightly more triangular in outline in female; somewhat bulging in profile, slightly protruding ventrally; ventral margin weakly convex in anterior view with dorsal margin of mandible weakly curved, mandibles weakly deflected, exposing part of labrum; base of mandible not expanded ventrally to form a basal tooth or lobe. Malar sulcus distinctly impressed throughout, deeper near eye. Antenna with 38–40 flagellomeres. Mesosoma 1.35–1.4 × longer than high. Pronotum laterally with vertical groove usually crenulate to rugulose dorsally and ventrally, weakly wrinkled medially posteriorad distinct vertical carina, carina weaker, evanescent dorsally and ventrally. Notaulus a short groove weakening posteriorly, extending nearly to level of anterior margin of tegula, widely separated from anterior margin, not margined anteriorly by carinae. Propodeum unsculptured, with a few weak carinulae along posterior margin. Fore wing 3RSa 1.25–1.4 × longer than 2RS; (RS+M)a usually weakly sinuate. T1 1.85–2.2 × wider at apex than at base, length 0.9–1.0 × apical width; smooth, unsculptured basally, striate to strigose over apical 0.6–0.7, more densely sculptured apical-medially; dorsal carina distinct, elevated basally, extending to apex but largely obscured by sculpture posteriorly, indicated primarily as lateral margin of very weakly elevated median area. Ovipositor (total length) 1.3 × longer than mesosoma; ovipositor sheath 0.8 × length of mesosoma. Color: Head similar in general color pattern to that of *Opius nablus* but dark facial spot sometimes (20%) more diffuse and not extending ventrally to epistomal sulcus; pale orbital ring in two specimens almost interrupted near torulus by traces of dark band extending to eye from dark patch on frons. Mesosoma black to dark red-brown except propleuron at least ventral-laterally, anterior declivity of mesoscutum near notaulus, subalar elevation, tegula and basal wing sclerite white to very pale yellow; most of mesoscutum, axilla, mesopleuron ventrally, and small spot on mesopleuron immediately dorsad mid coxa yellow; meso- and metanotum laterally, especially adjacent wing bases, varying from yellow to light brown, mesoscutum medially with narrow, faint to distinct dark median line in nearly all specimens. Metasomal terga dark reddish brown to black, with posterior margins of T3–T6 broadly white to hyaline and anterior margins of T4–T6 with broad white band medially; T7 white. Fore and mid tibiae and all femora pale yellow; hind tibia brown with basal 0.2 dark brown. Body length 3.3–3.9 mm; wing length 4.15–4.55 mm; mesosoma length 1.35–1.55 mm. Otherwise having all the characteristics described above for the *pipitae* species group.

##### Diagnosis.

This species is much lighter in color than *Opius stecki*. The mesosoma is completely dark in the latter species. The pronotum laterally also has a little more sculpture medially in *Opius pipitae* than in *Opius stecki*. *Opius pipitae* is very similar to *Opius townesi*, a previously described species for which no host information is available. *Opius townesi* is smaller, with significantly fewer flagellomeres (30), is slightly darker, without the pale orbital ring dorsally, and T1 is not as heavily sculptured. Although Fischer (1977) placed *Opius townesi* in the subgenus *Opius*, the labrum is exposed in the small but distinct gap between the ventral margin of the clypeus and the dorsal margin of the mandibles. *Opius townesi* is therefore included in the *pipitae* species group as defined here. *Opius townesi* was described from Maryland (USA).

##### Biology.

All specimens of *Opius pipitae* were reared from stem galls formed by an undescribed species in what may be an undescribed genus of Tephritidae attacking the asteracean *Montanoa frutescens*. One specimen of *Opius gabriellae* was reared from a species of *Neotephritis* infesting flower heads of his same host plant. Rate of parasitization by *Opius pipitae* was 50, 62.5, and 100% from the three sample sites, though overall the numbers were low with only 4 flies and 9 wasps reared.

##### Etymology.

The species is named for Pipit Godefroy, daughter of the senior author.

##### Remarks.

Color is somewhat variable in the type series. Some of the color differences are natural, but others are due to postmortem changes. There is natural variation in the degree of weak infumation on the propleuron and the presence of a faint median dark line of the mesoscutum. Postmortem changes include subcuticular darkening in some specimens and metasomal pale patches darkening from white to yellow.

#### Opius stecki

Wharton sp. n.

http://zoobank.org/A3A1B7FC-EDD3-4F18-8F03-61BBCAEB63FF

http://species-id.net/wiki/Opius_stecki

Figs 29 , 79–82

##### Type locality.

Guatemala, Deptartamento Zacapa, Sierra de las Minas.

##### Type material.

Holotype. Female (FSCA), first label, first line: GUATEMALA: Dept. Zacapa second line: Sierra de las Minas, San Lorenzo rd; third line: 1600–1700m; vic 15.07329, -89.68463; fourth line: 21–24 V 2010; Sutton, Steck, Skelley, fifth line: Monzon S.; oak forest Second label, first line: reared from galls *Polionota* n. sp. second line: (Diptera: Tephritidae) *ex Coreopsis* third line: *mutica* DC. (Compositae) fourth line: emerged late VI–VII 2010.

**Paratypes:** 2 females, same data as holotype (FSCA, TAMU).

##### Description.

*Female*. Eyes in dorsal view very slightly bulging beyond temples, temples very weakly receding. Clypeus 1.9–2.1 × wider than high, very weakly punctate throughout; weakly triangular in outline; weakly bulging, nearly flat in profile, slightly protruding ventrally; ventral margin weakly concave in anterior view with dorsal margin of mandibles nearly straight, very weakly deflected, narrowly exposing part of labrum; base of mandible slightly extended ventrally, though not developed as a discrete basal tooth, malar space thus a little shorter relative to basal width of mandible compared to *Opius pipitae*. Malar sulcus present, weak. Antenna broken in all specimens. Mesosoma 1.45–1.55 × longer than high. Pronotum laterally with vertical groove finely sculptured dorsally and ventrally, weakly wrinkled to smooth medially posteriorad distinct vertical carina, carina weaker, evanescent dorsally and ventrally. Notaulus a short groove weakening posteriorly, not as discrete posteriorly nor as long as in *Opius pipitae*, widely separated from anterior margin, not margined anteriorly by carinae. Propodeum largely smooth, polished, with weak, irregular sculpturing over posterior 0.4 in one paratype. Fore wing 3RSa 1.4 × longer than 2RS; (RS+M)a very weakly sinuate. T1 1.9–2.0 × wider at apex than at base, length 0.9–1.1 × apical width; smooth, unsculptured basally and laterally, very weakly rugulose, nearly smooth (paratype) to strigose over apical 0.6–0.7 (holotype); dorsal carina distinct, elevated basally, converging, extending almost to spiracle in paratype, stronger, distinct over basal 0.7–0.8 in holotype. Ovipositor (total length) approximately 1.2 × longer than mesosoma. Color: Head almost entirely black; clypeus varying from mostly black with ventral 0.3–0.4 yellow-orange to largely yellow-orange with small black spot mid-dorsally; base of mandible yellow, narrow band on malar space between clypeus and mandible whitish yellow; palps and remaining mouthparts white. Mesosoma entirely black. Metasoma black, T3–T6 with brownish posterior margins. Legs mostly yellow, nearly white basal-ventrally, tarsi dark brown, except fore basitarsis yellow, hind tibia dark brown over basal 0.15, brown over apical 0.4 fading to yellow medially. Body length 2.7–3.3 mm; wing length 3.8 mm; mesosoma length 1.15–1.4 mm. Otherwise having all the characteristics described above for the *pipitae* species group.

**Figures 29–32.**
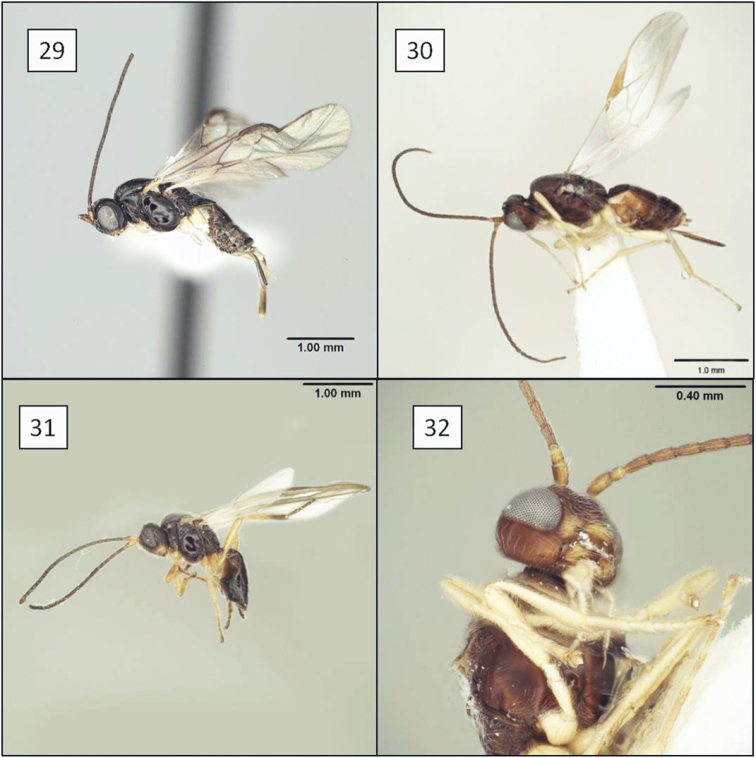
*Opius* spp., habitus and face. **29** *Opius stecki* Wharton, sp. n. **30** *Opius taramegillae* Wharton, sp. n. **31** *Opius yoderi* Wharton, sp. n. **32** *Opius taramegillae* face, ventral view.

##### Diagnosis.

This species is much darker in color than *Opius pipitae*. The head is completely dark above while the mesoscutum is extensively pale in *Opius pipitae* and is completely dark in *Opius stecki*. The ovipositor is also slightly shorter in *Opius stecki* and the mandibles not quite as deflected ventrally.

##### Biology.

The type series was reared from stem galls made by an apparently undescribed species of *Polionota* (Tephritidae) on the asteracean *Coreopsis mutica* DC.

##### Etymology.

This species is named for Gary Steck, one of the collectors of the sample that yielded the type series, for his contributions to our understanding of host relationships.

##### Remarks.

One of the specimens was sacrificed for sequencing; the morphological description is based on the two remaining specimens, which differed in several features. The paratype has weak sculpture on the propodeum posteriorly, unlike the holotype, though the propodeum is still largely polished and unsculptured as it is in most of the species described in this paper. Fore wing 2CUb arises more anteriorly on the distal margin of the 1st subdiscal cell in the holotype than in the paratype, though distinctly above the middle in both.

#### Opius taramegillae

Wharton sp. n.

http://zoobank.org/1DED8B0E-8698-4BBD-AE49-A47869A9FAAC

http://species-id.net/wiki/Opius_taramegillae

Figs 30 , 32 , 83–86

##### Type locality.

Mexico, Morelos, Parque Lago de Zempoala.

##### Type material.

Holotype. Female (UNAM), first label, first line: MEXICO: Morelos, Parque second line: Lag. de Zempoala, clear- third line: ing at entrance, 9–11. fourth line: VIII.1989, A.L.Norrbom Second label, first line: reared ex. stems second line: Barkleyanthus salici- third line: folius (H.B.K.) H. third line: Robins. & Brett. (89M1) fourth line: prob. ex. Paroxyna sp. Third label, first line: ALN second line: 34A.

##### Description.

*Female*. Eyes in dorsal view not bulging beyond temples; eye about 1.1–1.2 × longer than temple in dorsal view; 1.7 × longer than temple in lateral view. Vertex and frons densely setose. Face and frons smooth, polished. Clypeus somewhat crescentic, ventral margin strongly protruding in lateral view, without horn or spine-like protrusions, sharp, truncate in anterior view; labrum broadly exposed. Malar space slightly shorter than basal width of mandible, malar sulcus complete, deeply incised throughout. Mandible with dorsal margin not deflected, with distinct basal lobe ventrally, apical teeth not twisted. Occipital carina completely absent. Antenna with 26 flagellomeres; first flagellomere about 2.1–2.2 × longer than wide, 0.9 × length of second. Maxillary palp nearly as long as height of head. Pronotum dorsally narrow, with large pronope; laterally without vertical carina adjacent median vertical groove, groove narrow, discrete over dorsal 0.2, otherwise, broad, shallow, weakly indicated. Mesoscutum with deep, nearly vertical anterior declivity; notaulus and supramarginal carina completely absent; mesoscutum completely, uniformly densely covered with short, white, mostly decumbent setae; midpit narrowly elliptical, long, extending anteriorly more than half length of disc from posterior margin. Precoxal sulcus absent. Metapleuron unsculptured medially; median pit adjacent anterior margin and dorsal pit at posterior margin both relatively small, largely obscured by setae. Propodeal spiracle closer to anterior than posterior margin; propodeum densely setose and weakly punctate throughout, with some weakly rugulose sculpture adjacent posterior margin, otherwise unsculptured. Hind tibia without basal carina. Fore wing stigma wedge-shaped, discrete distally, r arising from basal 0.35; 3RSa 1.5 × longer than 2RS, 2nd submarginal cell strongly narrowing distally, 2r-m equal in length to 2Ma; 3RSb extending nearly to apex of wing; m-cu distinctly postfurcal; 2CUb arising distinctly below middle of distal margin of 1st subdiscal cell, 1st subdiscal cell closed apically; 1cu-a interstitial; distance between anal vein and ventral wing margin equal to about 1.5 × width of anal vein. Hind wing RS largely absent; m-cu present, extending nearly to wing margin as a posteriorly weakening crease. S1 short, barely visible in lateral view; T1 with laterope but without dorsope; dorsal carinae distinct basally on either side of deep basal depression, difficult to distinguish from surrounding strigose sculpture over posterior 0.6; T1 1.15 × longer than apical width; apex 2.0 × wider than base. T2 and following terga unsculptured. Base of ovipositor well-concealed, ovipositor (total length) very approximately 1.5 × longer than mesosoma; ovipositor sheath 0.9 × length of mesosoma. Color: Brown, T2+3 and tegula yellow-brown; clypeus, lower face and malar space adjacent clypeus, and mandible (except apical teeth) yellow; palps white, legs pale yellow, almost white; wings hyaline. Body length 2.5 mm; wing length 2.7 mm; mesosoma length 1.05 mm.

##### Diagnosis.

This species can be recognized by the combination of the complete absence of a notaulus, complete absence of an occipital carina, presence of a densely setose mesoscutum with long, narrow midpit, and presence of a basal lobe or tooth ventrally on the mandible. *Opius taramegillae* is most similar to the equally densely setose *Opius cosa* (Fischer), comb. n., but the coxae are distinctly darker in *Opius cosa* and the venation is somewhat different, most notably with the 1st subdiscal cell open apically in *Opius cosa*.

##### Biology.

The holotype was reared from stems of the asteracean *Barkleyanthus salicifolius*, the same host plant and plant part that yielded one of the reared series of *Opius nympha*. The probable fly host is a species of *Campiglossa* since two flies belonging to this genus were reared from the same sample of stems that produced the wasp. As noted above, *Campiglossa* is currently treated as a senior synonym of *Paroxyna*.

##### Etymology.

This species is named for Tara Megill, daughter of the senior author.

##### Remarks.

This species keys to *Bracanastrepha (Bracanastrepha)* in Fischer (1977) due to the complete loss of the occipital carina in combination with the distinct midpit on the mesoscutum. Wharton (1988, 1997a) placed *Bracanastrepha* s.s. as a synonym of *Utetes*, but also noted that several species placed in *Bracanastrepha* by Fischer (1977) did not share the tibial carination characteristic of the type species of both *Utetes* and *Bracanastrepha*. These remaining species are currently included in *Opius* s.l., as explained most recently by Wharton et al. (2012). Hence, both *taramegillae* and *cosa* are placed in *Opius* until this portion of the Opiinae can be more thoroughly revised. The classification presented in the key by Li et al. (2013) does not cover these New World groups.

*Opius taramegillae* and *Opius cosa* represent another distinctive species group within *Opius* s.l., most easily differentiated from all the others treated here by the complete absence of the occipital carina and the long, deep midpit of the mesoscutum. As in *Opius nympha* and *Opius yoderi*, the distal abscissa of fore wing CU arises posteriorad the middle of the 1st subdiscal cell, a common feature of opiines in general, but unusual among those opiines attacking stem and flower-infesting tephritids in the New World.

Although *Opius taramegillae* is known only from the holotype, the description seems warranted to highlight yet another distinctive group of tephritid parasitoids within the Opiinae. It will be useful to obtain additional reared material to verify *Campiglossa* as the normal host for this species and to develop a better understanding of host plant relationships.

#### Opius yoderi

Wharton sp. n.

http://zoobank.org/F210B1D0-8399-4D6D-9EA0-B797AA7FA946

http://species-id.net/wiki/Opius_yoderi

Figs 31 , 87–90

##### Type locality.

Mexico, Morelos, Parque Lago de Zempoala.

##### Type material.

Holotype. Female (UNAM), first label, first line: MEXICO: Morelos, Parque second line: Lag. de Zempoala, clear- third line: ing at entrance, 9–11. fourth line: VIII.1989, A.L.Norrbom Second label, first line: reared ex. flowers second line: *Dahlia imperialis* third line: Roezl. ex Ort. (89M12) Third label, first line: ALN second line: 39.

**Paratypes:** 2 females, same data as holotype (USNM, TAMU).

##### Description.

*Female*. Eyes in dorsal view very slightly bulging beyond temples, temples very weakly receding; eye about 1.5–1.7 × longer than temple in dorsal view; 2.3–2.5 × longer than temple in lateral view. Face and vertex moderately to densely setose; frons bare. Clypeus tall, somewhat oval, 1.3 × broader than tall, ventral margin not protruding in lateral view, without horn or spine-like protrusions, sharp, convex in anterior view; labrum completely concealed. Malar space about equal to basal width of mandible, malar sulcus weak, not deeply incised. Mandible with dorsal margin not deflected, without basal lobe ventrally, apical teeth not twisted. Occipital carina absent middorsally, widely separated from hypostomal carina at base of mandible. Antenna with 19–21 flagellomeres; first flagellomere about 2.5–3.0 × longer than wide, 1.05–1.25 × length of second. Maxillary palp shorter than height of head. Mesosoma 1.4 × longer than high, 1.95 × longer than wide, 1.4 × higher than wide. Pronotum dorsally narrow, with large pronope; laterally bare, polished, with well-developed vertical carina all along anterior margin of median vertical groove. Mesoscutum with anterior declivity about as in *taramegillae*; notaulus and supramarginal carina completely absent; mesoscutum completely, uniformly densely covered with short, white, mostly decumbent setae; midpit absent. Precoxal sulcus distinctly though shallowly impressed, unsculptured; extending from anterior margin in two specimens, confined to middle 0.3 of mesopleuron in third specimen, never reaching base of coxa. Metapleuron unsculptured medially, sparsely setose; median pit adjacent anterior margin and dorsal pit at posterior margin both relatively small, not connected by deep sulcus. Propodeal spiracle closer to anterior than posterior margin; propodeum sparsely setose, densely granular-rugulose throughout. Hind tibia without basal carina. Fore wing stigma narrowly wedge-shaped, merging imperceptibly with R1 distally, r arising from basal 0.3; 3RSa 1.4–1.5 × longer than 2RS, 2nd submarginal cell very strongly narrowing distally; 3RSb bowed, extending nearly to apex of wing; m-cu very widely antefurcal; 2CUb arising distinctly below middle of distal margin of 1st subdiscal cell, 1st subdiscal cell open apically, 2cu-a completely absent; 1cu-a weakly postfurcal; distance between anal vein and ventral wing margin slightly less than width of anal vein. Hind wing with RS and 2M equally developed as very weakly pigmented creases extending to wing margin, m-cu slightly weaker, extending at least 0.5 distance to wing margin. S1 short but visible, 0.15–0.2 × length of T1; T1 with laterope but without dorsope; largely smooth basally, striate over posterior 0.7; dorsal carinae distinct basally on either side of broad, shallow basal depression, usually extending nearly to posterior margin, less distinct posteriorly amongst striate sculpture; T1 0.9–1.1 × longer than apical width; apex 2.1–2.25 × wider than base. T2 and following terga unsculptured. Ovipositor (total length) approximately 0.8–0.9 × length of mesosoma; ovipositor sheath approximately 0.3–0.4 × length of mesosoma. Color: Dark brown, T2+3 light brown, tegula, clypeus, ventral margin of face from anterior tentorial pit through lower part of malar space, mandible (except apical teeth), palps, and most of legs yellow to very pale yellow; tarsi and apical 0.3–0.4 of hind tibia variously brown; wings hyaline. Body length 1.8–2.4 mm; wing length 2.6–2.7 mm; mesosoma length 0.95–0.75 mm.

##### Diagnosis.

*Opius yoderi* is readily differentiated from all other species treated here by the combination of widely antefurcal fore wing m-cu and densely setose mesoscutum. *Opius taramegillae* also has a densely setose mesoscutum but has a long, deep midpit that is lacking in *Opius yoderi*. *Opius yoderi* has a shorter, broader T1 than *Opius simplex* Fischer from Costa Rica and *Opius columbicus* Fischer from Colombia, the two previously described species that it most closely resembles. Further, T1–3 are darker in *Opius yoderi* than in *Opius simplex* and the second submarginal cell is more strongly narrowed distally in *Opius yoderi* than in *Opius columbicus*.

##### Biology.

All members of the type series were reared from flowers of the asteracean *Dahlia imperialis* but no flies were reared from this sample. Flowers of this same plant, collected from the same general locality two years later, yielded two different opiines: *Doryctobracon anneae* and *Opius danielsae*, reared from the tephritids *Gymnocarena mexicana* and *Paracantha trinotata* respectively. These flies and wasps are significantly larger than *Opius yoderi*, and we predict a smaller tephritid is more likely the host to this particular wasp.

##### Etymology.

This species is named for Matt Yoder for his contributions to databasing in general and more specifically for considerable facilitation of the work done here and for related contributions to biodiversity of parasitic Hymenoptera.

##### Remarks.

This species runs to *Opius (Opius)* in the keys and classification of Fischer (1977, 1983) due to the concealed labrum and absence of a mesoscutal midpit and absence of a sculptured precoxal sulcus. Alternatively, *Opius yoderi* would be placed in *Phaedrotoma* in the classification of Li et al. (2013), though with minor difficulty since the venation is more similar to that of *Rhogadopsis*. Fischer (1999) presented a revised classification, including the description of a new genus, *Neotropopius* Fischer. Only one species has ever been included in *Neotropopius*, the type species, *Neotropopius hirtithorax* Fischer. *Opius yoderi* fits the characterization of *Neotropopius* presented by Fischer (1999). *Neotropopius* was treated as a synonym of *Phaedrotoma* by Li et al. (2013) even though the presence of a median keel on the propodeum of the type species would seem to argue for a placement in *Rhogadopsis*.

The type series of *Opius yoderi* is somewhat variable in features such as the length of the precoxal sulcus and the shape and sculpture of T1. The holotype also has slightly more extensive pale coloration on the lower face and malar space than in the paratypes.

**Figures 33–36.**
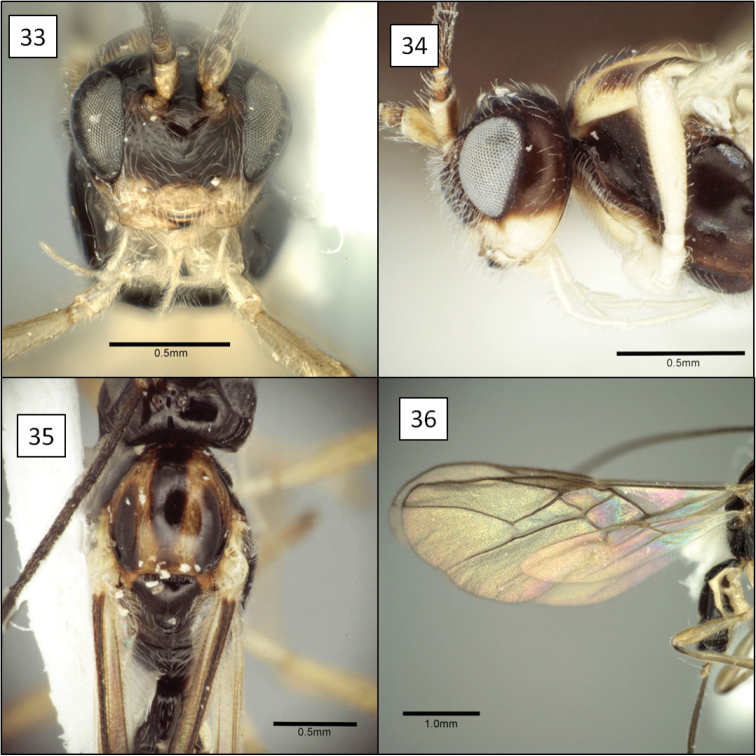
*Opius baderae* Wharton, sp. n. **33** face, anterior view **34** head, lateral view **35** mesoscutum, dorsal view **36** wings.

**Figures 37–40.**
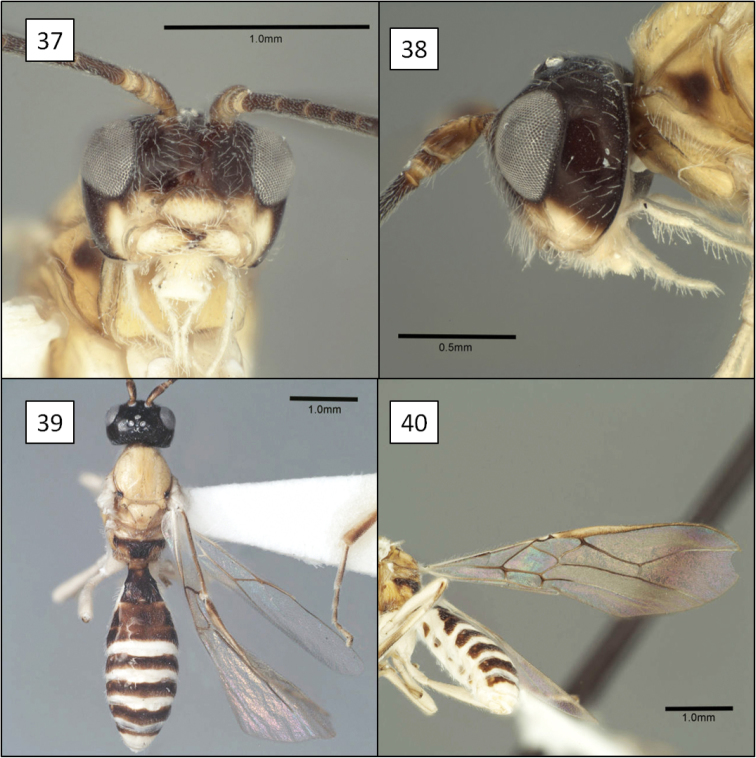
*Opius baeblus* Wharton, sp. n. **37** face, anterior view **38** head, lateral view **39** dorsal habitus **40** wings.

**Figures 41–46.**
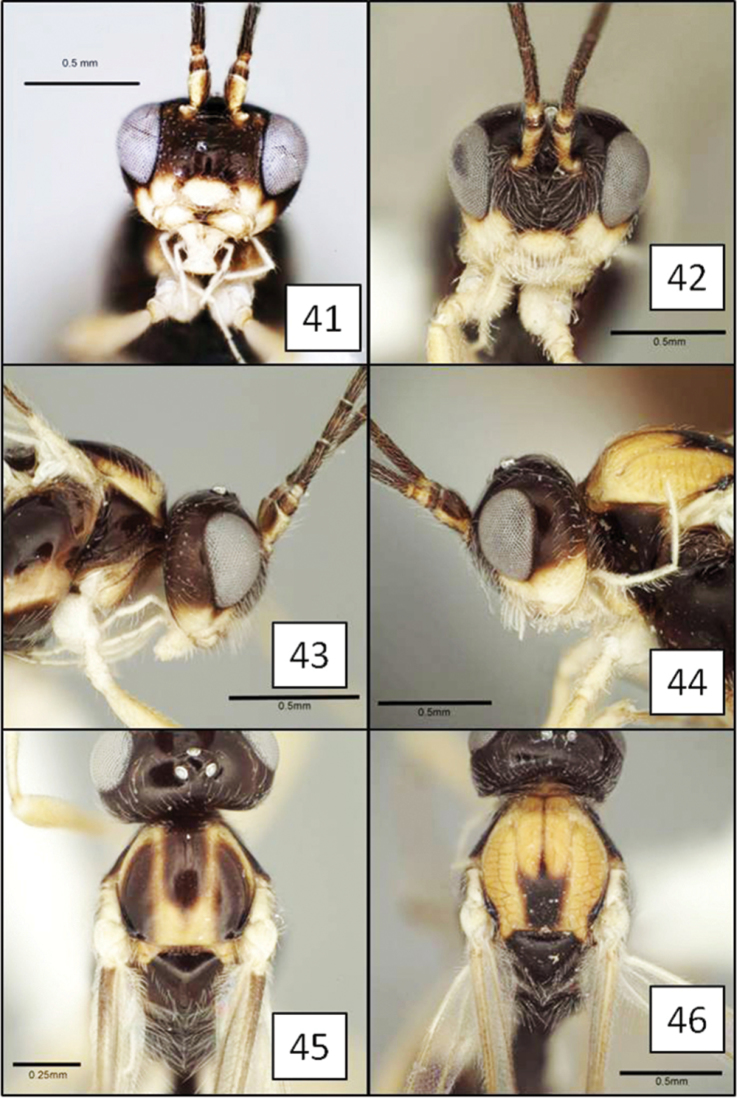
*Opius* spp. **41** *Opius cablus* Wharton, sp. n., face, anterior view **42** *Opius dablus* Wharton, sp. n., face, anterior view **43** *Opius cablus* head, lateral view **44** *Opius dablus* head, lateral view **45** *Opius cablus* mesosoma, dorsal view **46** *Opius dablus* mesosoma, dorsal view.

**Figures 47–50.**
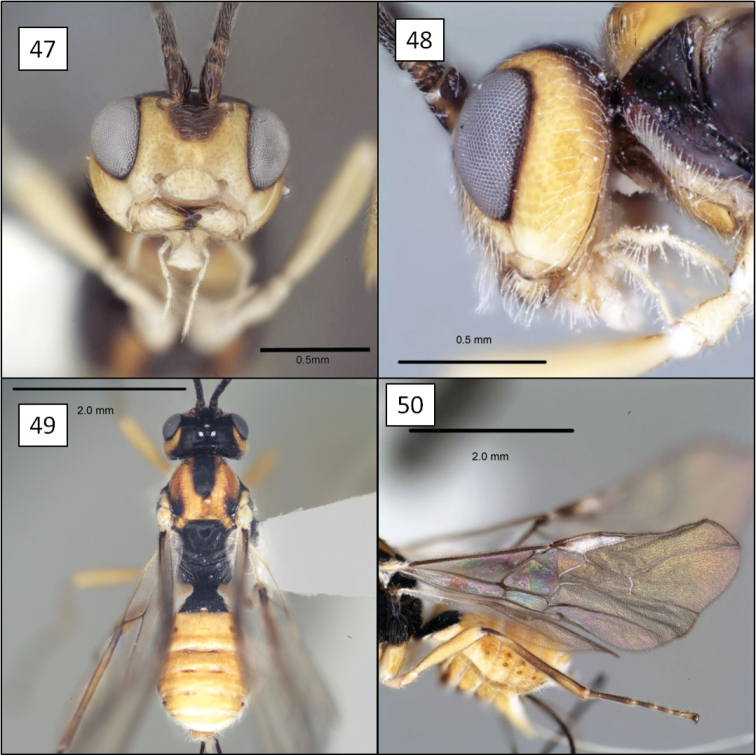
*Opius danielsae* Wharton, sp. n. **47** face, anterior view **48** head, lateral view **49** dorsal habitus **50** wings.

**Figures 51–54.**
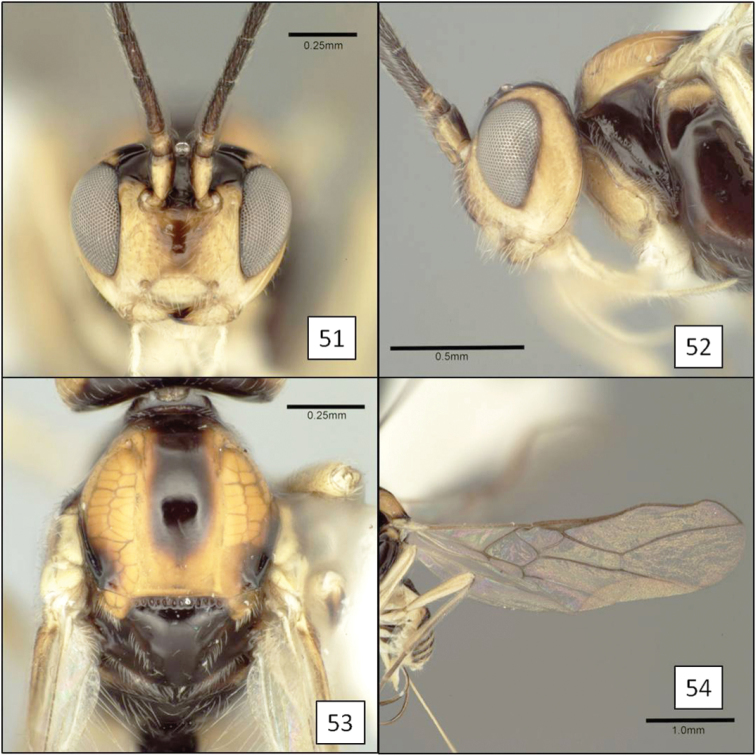
*Opius gabriellae* Wharton, sp. n. **51** face, anterior view **52** head, lateral view **53** mesosoma, dorsal view **54** wings.

**Figures 55–58.**
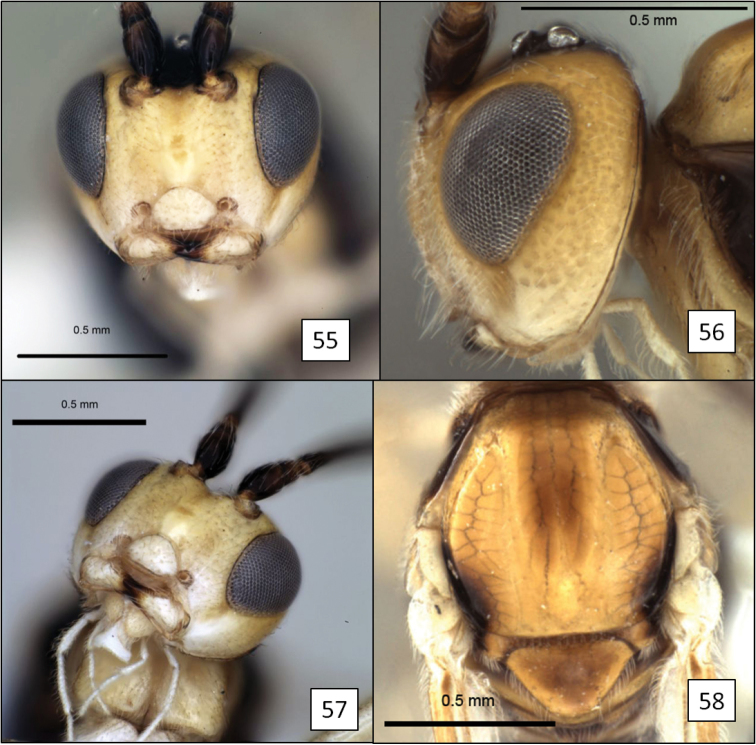
*Opius godfrayi* Wharton, sp. n. **55** face, anterior view **56** head, lateral view **57** face tilted to show labrum narrowly exposed **58** mesoscutum, dorsal view.

**Figures 59–62.**
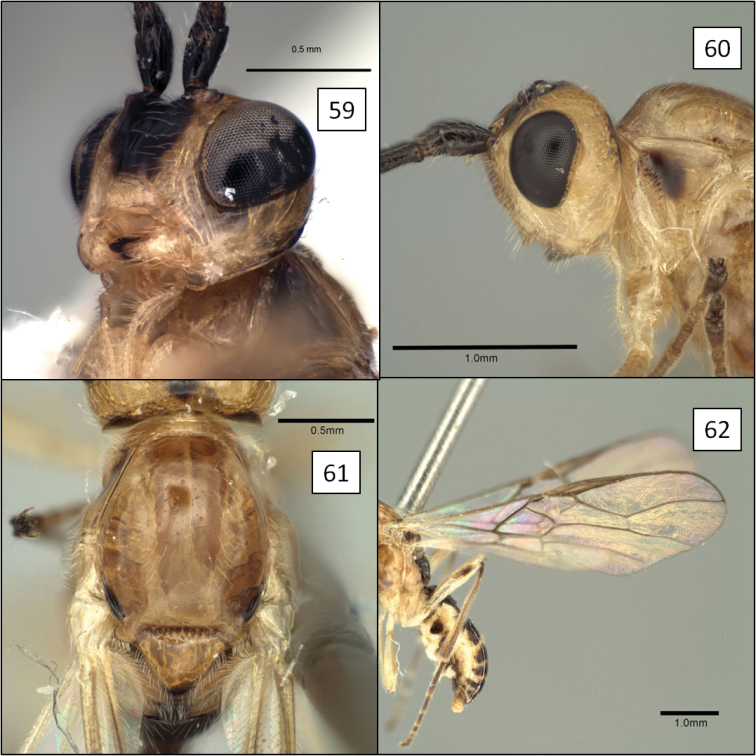
*Opius marshi* Wharton, sp. n. **59** face, anterior-lateral view **60** head, lateral view **61** mesoscutum, dorsal view **62** wings.

**Figures 63–66.**
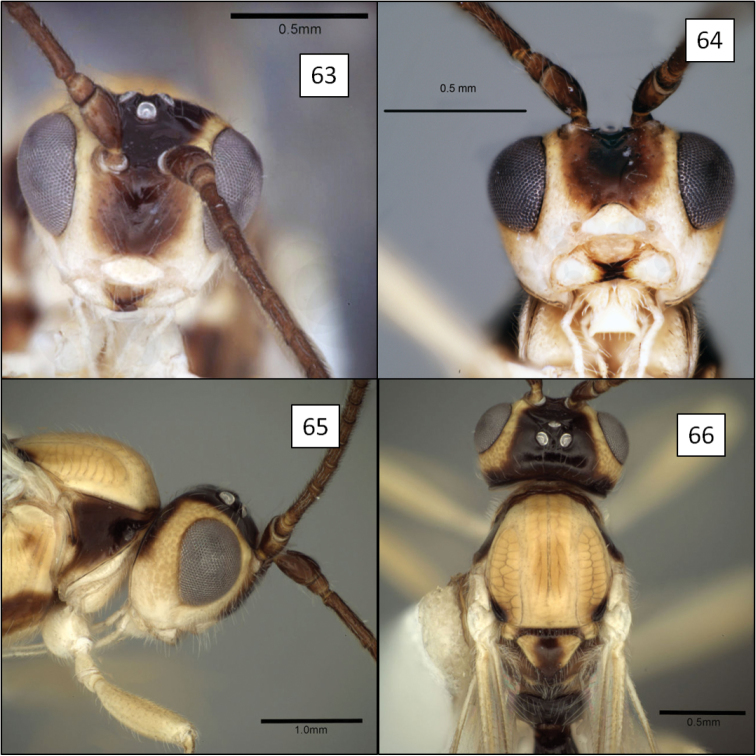
*Opius nablus* Wharton, sp. n. **63** face, anterior view **64** face, rotated to show broadly exposed labrum **65** head, lateral view **66** mesosoma, dorsal view.

**Figures 67–70.**
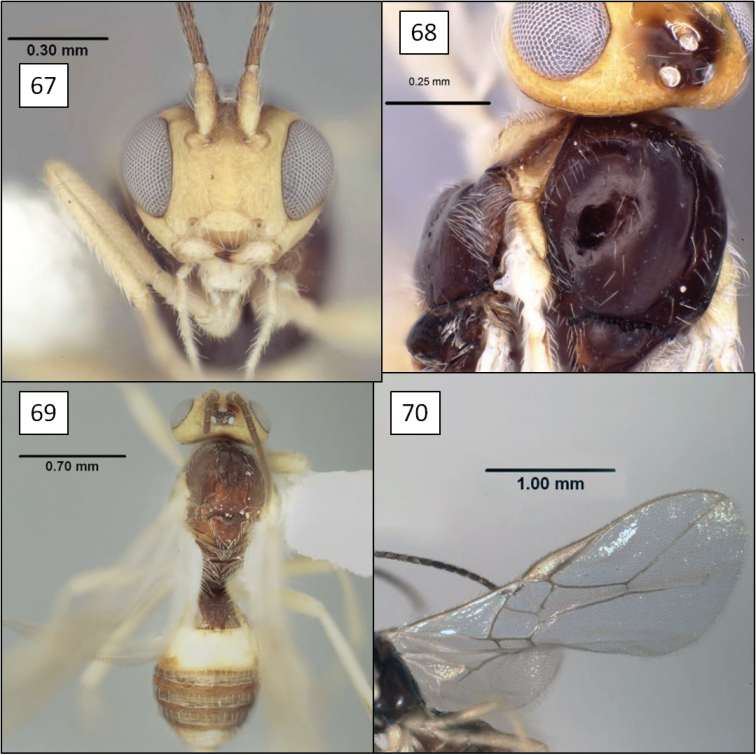
*Opius nympha* Fischer. **67** face, anterior view **68** head and mesosoma, lateral view **69** dorsal habitus **70** wings.

**Figures 71–74.**
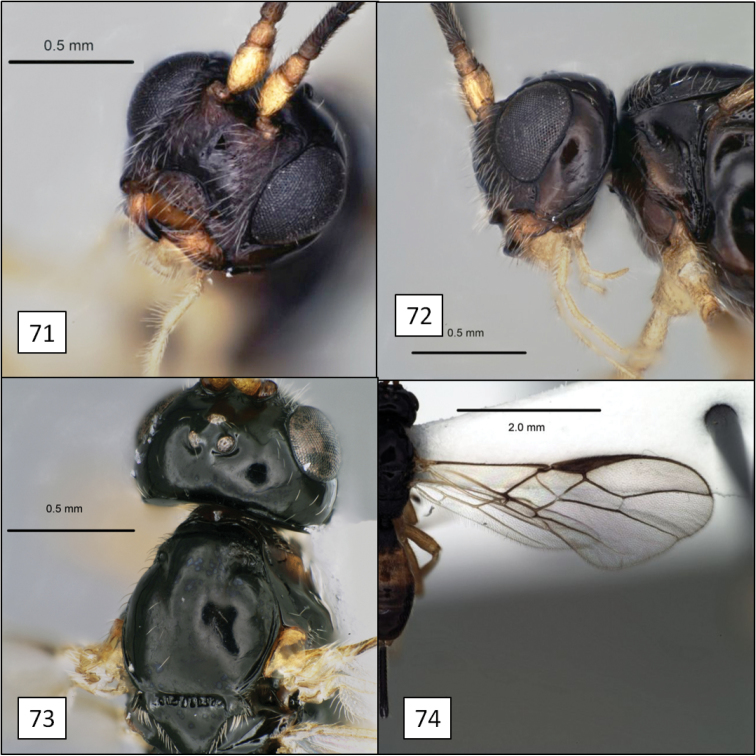
*Opius peleus* Fischer. **71** face, anterior view **72** head, lateral view **73** mesoscutum, dorsal view **74** wings.

**Figures 75–78.**
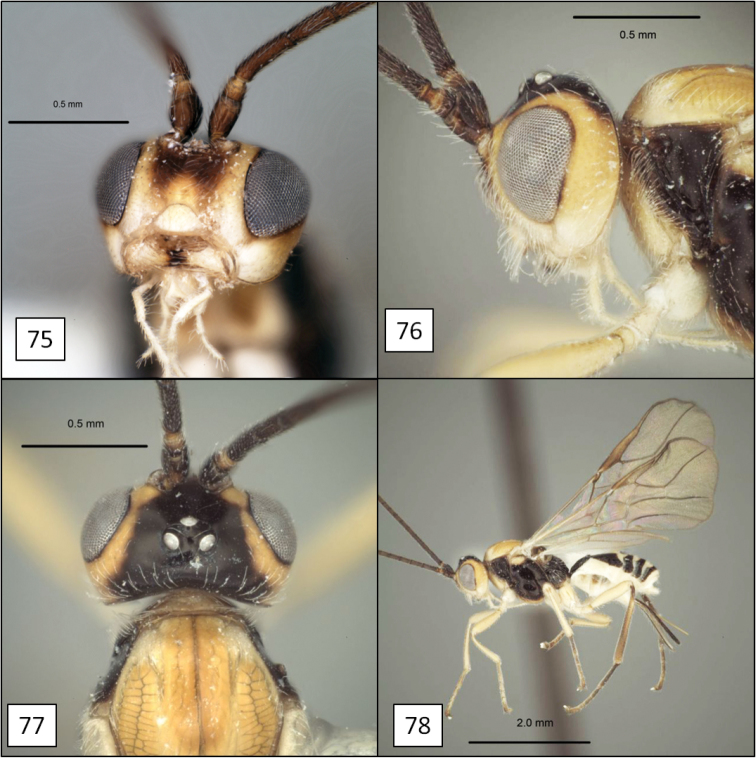
*Opius pipitae* Wharton, sp. n. **75** face, anterior view **76** head, lateral view **77** head and mesoscutum, dorsal view **78** wings.

**Figures 79–82.**
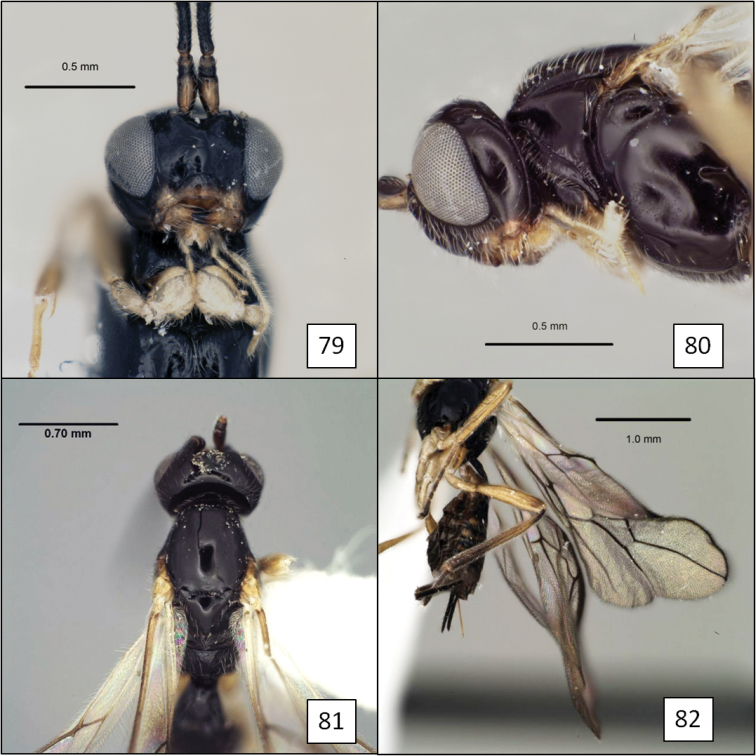
*Opius stecki* Wharton, sp. n. **79** face, anterior view **80** head, lateral view **81** mesosoma, dorsal view **82** wings.

**Figures 83–86.**
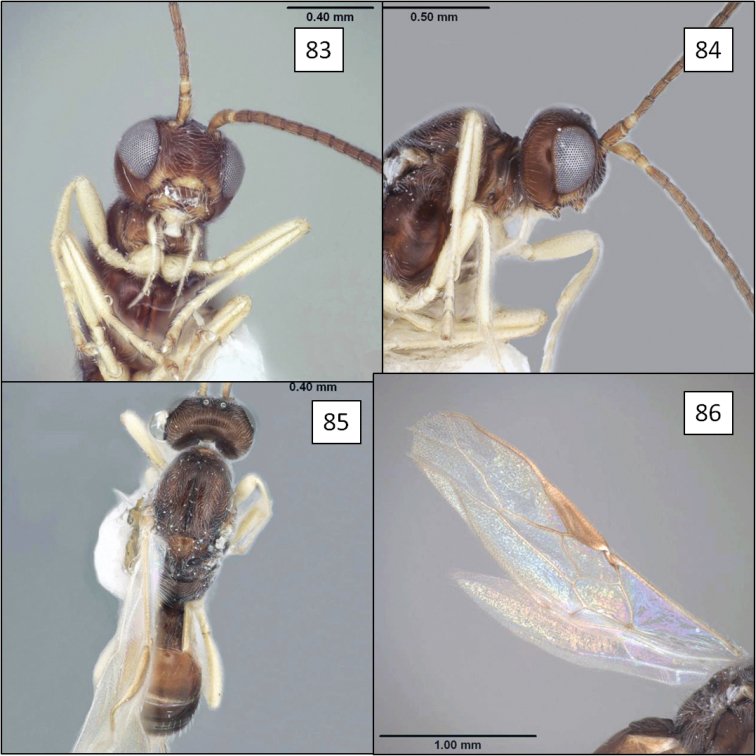
*Opius taramegillae* Wharton, sp. n. **83** face, anterior view **84** head, lateral view **85** dorsal habitus **86** wings.

**Figures 87–90.**
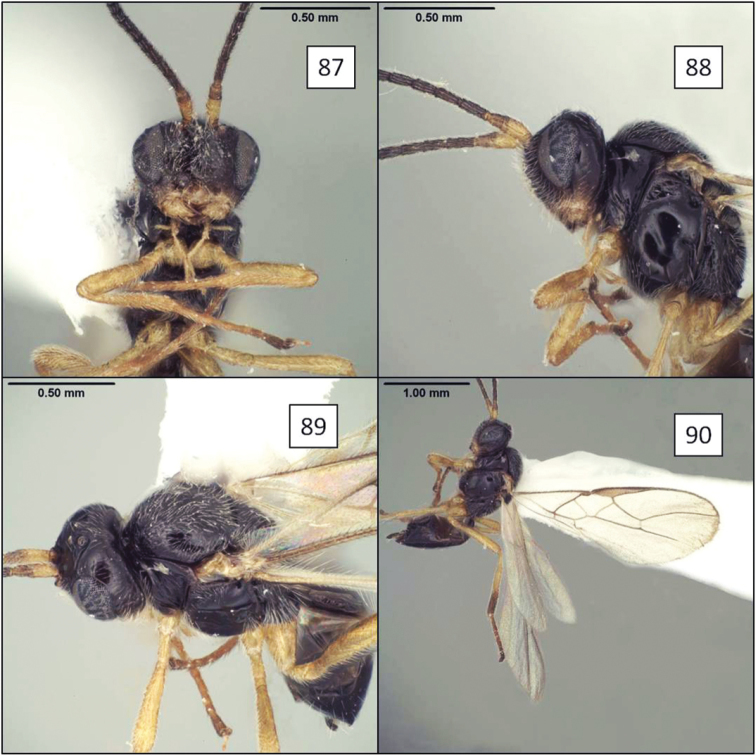
*Opius yoderi* Wharton, sp. n. **87** face, anterior view **88** head and mesosoma, lateral view **89** dorsal-oblique habitus **90** wings.

## Supplementary Material

XML Treatment for Doryctobracon

XML Treatment for Doryctobracon anneae

XML Treatment for Opius baderae

XML Treatment for Opius baeblus

XML Treatment for Opius cablus

XML Treatment for Opius dablus

XML Treatment for Opius danielsae

XML Treatment for Opius gabriellae

XML Treatment for Opius godfrayi

XML Treatment for Opius marshi

XML Treatment for Opius nablus

XML Treatment for Opius nympha

XML Treatment for Opius peleus

XML Treatment for Opius pipitae

XML Treatment for Opius stecki

XML Treatment for Opius taramegillae

XML Treatment for Opius yoderi

## References

[B1] Aluja M López M Sivinski J (1998) Ecological evidence for diapause in four native and one exotic species of larval-pupal fruit fly (Diptera: Tephritidae) parasitoids in tropical environments. Annals of the Entomological Society of America 91: 821-833.

[B2] Belokobylskij SA Taeger A van Achterberg C Haeselbarth E Riedel M (2003) Checklist of the Braconidae of Germany (Hymenoptera). Beitraege zur Entomologie 53: 341-435.

[B3] Clancy DW (1950) Notes on parasites of tephritid flies. Proceedings of the Hawaiian Entomological Society 14: 25-26.

[B4] Denlinger DL (1986) Dormancy in tropical insects. Annual Review of Entomology 31: 239-264. doi: 10.1146/annurev.en.31.010186.001323 3510585

[B5] Duan JJ Messing RH (1999) Effects of origin and experience on patterns of host acceptance by the opiine parasitoid *Diachasmimorpha tryoni*. Ecological Entomology 24: 284-291. doi: 10.1046/j.1365-2311.1999.00206.x

[B6] Duan JJ Purcell MF Messing RH (1997) Ovipositional responses of three opiine fruit fly parasitoids (Hymenoptera: Braconidae) to gall-forming tephritids (Diptera: Tephritidae). Biological Control 9: 81-88. doi: 10.1006/bcon.1997.0526

[B7] Enderlein G (1920) Zur Kenntnis aussereuropaischer Braconiden. Archiv fuer Naturgeschichte 11: 51-224.

[B8] Fischer M (1967) Zusammungfassung der neotropischen Opiinae mit Ausschluss der Gattung *Opius* Wesm. (Hymenoptera, Braconidae). Beitraege zur Neotropischen Fauna 5: 1-21. doi: 10.1080/01650526709360393

[B9] Fischer M (1968) Die neotropischen Genus *Opius*-Arten der Sektion C (Hymenoptera, Braconidae). Polskie Pismo Entomologiczne 38: 33-139.

[B10] Fischer M (1970) Nearktische Opiinae aus der Sammlung Townes (Hymenoptera, Braconidae). Polskie Pismo Entomologiczne 40: 763-827.

[B11] Fischer M (1971) Index of Entomophagous Insects. Hymenoptera Braconidae. World Opiinae. Le François, Paris, 189 pp.

[B12] Fischer M (1972) Hymenoptera Braconidae (Opiinae I). Das Tierreich 91: 1-620.

[B13] Fischer M (1977) Hymenoptera Braconidae (Opiinae II-Amerika). Das Tierreich 96: 1-1001.

[B14] Fischer M (1979) Zur Kenntnis der Artenvielfalt bei den Opiinen-Wespen in der neotropischen Region (Hymenoptera, Braconidae, Opiinae). Polskie Pismo Entomologiczne 49: 227–297.

[B15] Fischer M (1983) Neubeschreibungen von neotropischen Opiinae aus den Gattungen *Desmiostoma*, *Bracanastrepha* und *Opius* (Hymenoptera, Braconidae). Entomologische Abhandlungen Staatliches Museum fuer Tierkunde in Dresden 47: 65-94.

[B16] Fischer M (1987) Hymenoptera Opiinae III - aethiopische, orientalische, australische und ozeanische Region. Das Tierreich 104: 1-734.

[B17] Fischer M (1999) Zur Evolution und zum System der *Opius*-verwandten Gattungen der Unterfamilie Opiinae mit einer erweiterten Aufteilung dieses Gattungs-Komplexes (Hymenoptera, Bracondiae, Opiinae). Linzer Biologische Beitraege 31: 277-336.

[B18] Karlsson D Ronquist F (2012) Skeletal morphology of *Opius dissitus* and *Biosteres carbonarius* (Hymenoptera: Braconidae), with a discussion of terminology. PLoS ONE 7(4): e32573. doi: 10.1371/journal.pone.0032573 22558068 PMC3340384

[B19] Korneyev VA (1999) Phylogeny of the subfamily Tephritinae: Relationships of the tribes and subtribes. In: Aluja M Norrbom AL (Eds) Fruit flies (Tephritidae): Phylogeny and evolution of behavior. CRC Press, Boca Raton, 549-580. doi: 10.1201/9781420074468.sec6

[B20] Li X-Y van Achterberg C Tan J-C (2013) Revision of the subfamily Opiinae (Hymenoptera, Braconidae) from Hunan (China), including thirty-six new species and two new genera. ZooKeys 268: 1-186. doi: 10.3897/zookeys.268.4071 PMC3592199 23653521

[B21] Marsh PM (1974) New combinations and new synonyms in North American Braconidae (Hymenoptera). Proceedings of the Entomological Society of Washington 76: 285-289.

[B22] Marsh PM (1979) Family Braconidae. In: Krombein KV Hurd PD Smith DR Burks BD (Eds) Catalog of Hymenoptera in America North of Mexico Volume 1 Smithsonian Institution Press, Washington, D.C., 144-295.

[B23] Norrbom AL (2006) New species and host records for *Gymnocarena* (Diptera, Tephritidae). In: Merz B (Ed) Phylogeny, taxonomy, and biology of tephritoid flies (Diptera, Tephritoidea). Proceedings of the “3rd Tephritoid Taxonomist’s Meeting”, Geneva, 19–24 July 2004. Instrumentas Biodiversitatis 7: 217–226.

[B24] Norrbom AL (2010) Tephritidae (fruit flies, moscas de frutas). In: Brown BV Borkent A Cumming JM Wood DM Woodley NE Zumbado MA (Eds) Manual of Central American Diptera, volume 2 NRC Research Press, Ottawa, 909-954.

[B25] Norrbom AL Sutton BD Steck GJ Monzón J (2010) New genera, species and host plant records of Nearctic and Neotropical Tephritidae (Diptera). Zootaxa 2398: 1–65.

[B26] Pemberton CE Willard HF (1918) A contribution to the biology of fruit-fly parasites in Hawaii. Journal of Agricultural Research 25: 419-467.

[B27] Seltmann KC Yoder MJ Mikó I Forshage M Bertone MA Agosti D Austin AD Balhoff JP Borowiec ML Brady SG Broad GR Brothers DJ Burks RA Buffington M L Campbell HM Dew KJ Ernst AF Fernández-Triana JL Gates MW Gibson GAP Jennings JT Johnson NF Karlsson D Kawada R Krogmann L Kula RR Mullins PL Ohl M Rasmussen C Ronquist F Schulmeister S Sharkey MJ Talamas E Tucker E Vilhelmsen L Ward PS Wharton RA Deans AR (2012) A hymenopterists’ guide to the Hymenoptera Anatomy Ontology: utility, clarification, and future directions. Journal of Hymenoptera Research 27: 67-88. doi: 10.3897/jhr.27.2961

[B28] Sharkey MJ Wharton RA (1997) Morphology and terminology. In: Wharton RA Marsh PM Sharkey MJ (Eds) Manual of the New World Genera of the Family Braconidae (Hymenoptera). The International Society of Hymenopterists, Washington, D.C., 19-37.

[B29] Tobias VI (1977) The genus *Opius* Wesm. (Hymenoptera, Braconidae) as parasites of fruit flies (Diptera, Tephritidae). Entomologicheskoe Obozrenie 56: 420-430.

[B30] Tobias VI (1998) Subfamily Opiinae. In: Ler AP (Ed) Key to the insects of Russian Far East. 4. Neuropteroidea, Mecoptera, Hymenoptera. 3. Hymenoptera (part). Dal’nauka, Vladivostok, 558-655.

[B31] Tropicos.org (2013) Missouri Botanical Garden. http://www.tropicos.org [accessed 27.II.2013]

[B32] Van Achterberg C (1997) Revision of the Haliday collection of Braconidae (Hymenoptera). Zoologische Verhandelingen 314: 1-115.

[B33] Van Achterberg C (2004) New Indo-Australian subgenera and species of the genera *Xynobius* Foerster and *Ademoneuron* Fischer (Hymenoptera: Braconidae: Opiinae). Zoologische Mededelingen 78: 313-329.

[B34] Van Achterberg C Salvo A (1997) Reared Opiinae (Hymenoptera: Braconidae) from Argentina. Zoologische Mededelingen Leiden 71: 189-214.

[B35] Walker AK Wharton RA (2011) A review of New World *Eurytenes* s. str. (Hymenoptera, Braconidae, Opiinae). Journal of Hymenoptera Research 20: 23-46.

[B36] Ward LA Wilson C Saenz C Harrell LK Steck GJ Wharton RA (2013) New host plant and distribution records of Tephritidae (Diptera) from Texas, with notes on parasitism of Tephritidae by Opiinae (Hymenoptera: Braconidae). Proceedings of the Entomological Society of Washington 115: 96-102. doi: 10.4289/0013-8797.115.1.96

[B37] Wharton RA (1983) Variation in *Opius hirtus* Fischer and discussion of *Desmiostoma* Foerster (Hymenoptera: Braconidae). Proceedings of the Entomological Society of Washington 85: 327–330.

[B38] Wharton RA (1987) Changes in nomenclature and classification of some opiine Braconidae (Hymenoptera). Proceedings of the Entomological Society of Washington 89: 61-73.

[B39] Wharton RA (1988) Classification of the braconid subfamily Opiinae (Hymenoptera). The Canadian Entomologist 120: 333-360. doi: 10.4039/Ent120333-4

[B40] Wharton RA (1997a) Generic relationships of opiine Braconidae (Hymenoptera) parasitic on fruit-infesting Tephritidae (Diptera). Contributions of the American Entomological Institute 30: 1-53.

[B41] Wharton RA (1997b) Subfamily Opiinae. In: Wharton RA Marsh PM Sharkey MJ (Eds) Manual of the New World Genera of the Family Braconidae (Hymenoptera). The International Society of Hymenopterists, Washington, D.C., 378-395.

[B42] Wharton RA (2006) The species of *Sternaulopius* Fischer (Hymenoptera: Braconidae, Opiinae) and the braconid sternaulus. Journal of Hymenoptera Research 15: 317-347.

[B43] Wharton RA (2009) Two new species of *Psyttalia* Walker (Hymenoptera, Braconidae, Opiinae) reared from fruit-infesting tephritid (Diptera) hosts in Kenya. ZooKeys 20: 349-377. doi: 10.3897/zookeys.20.99

[B44] Wharton RA Marsh PM (1978) New World Opiinae (Hymenoptera: Braconidae) parasitic on Tephritidae (Diptera). Journal of the Washington Academy of Sciences 68: 147-167.

[B45] Wharton RA Daniels S Shirley X Restuccia D (2013) An opiine Braconidae (Hymenoptera) reared from Richardiidae (Diptera) and recognition of a new species group of *Opius* s. l. ZooKeys 289: 65-101. doi: 10.3897/zookeys.289.4900 PMC3677391 23794854

[B46] Wharton R Ward L Miko I (2012) New neotropical species of Opiinae (Hymenoptera, Braconidae) reared from fruit-infesting and leaf-mining Tephritidae (Diptera) with comments on the *Diachasmimorpha mexicana* species group and the genera *Lorenzopius* and *Tubiformopius*. ZooKeys 243: 27-82. doi: 10.3897/zookeys.243.3990 PMC3697044 23818811

[B47] Wharton RA Yoder MJ (2012) Parasitoids of fruit-infesting Tephritidae. http://www.paroffit.org [accessed 9.V.2013]

[B48] Yoder M Dole K Deans A (2006) Introducing ‘mx’, a sharable digital workbench for systematic biologists. Proceedings of Taxonomic Database Working Group. http://www.tdwg.org/proceedings/article/view/38/0 [accessed 1 Sept 2009]

[B49] Yoder MJ Mikó I Seltmann K Bertone MA Deans AR (2010) A gross anatomy ontology for Hymenoptera. PLoS ONE 5(12): e15991. doi: 10.1371/journal.pone.0015991 21209921 PMC3012123

[B50] Yu DS Van Achterberg K Horstmann K (2005) World Ichneumonoidea 2004. Taxonomy, biology, morphology and distribution. Taxapad 2005. CD/DVD. Taxapad, Vancouver, www.taxapad.com

[B51] Yu DSK Van Achterberg C Horstmann K (2012) Taxapad 2012 - World Ichneumonoidea 2011. Taxonomy, biology, morphology and distribution. On USB Flash drive. www.taxapad.com, Ontario.

